# Benchmarking hybrid assembly approaches for genomic analyses of bacterial pathogens using Illumina and Oxford Nanopore sequencing

**DOI:** 10.1186/s12864-020-07041-8

**Published:** 2020-09-14

**Authors:** Zhao Chen, David L. Erickson, Jianghong Meng

**Affiliations:** Joint Institute for Food Safety and Applied Nutrition, Center for Food Safety and Security Systems, and Department of Nutrition and Food Science, University of Maryland, College Park, MD 20742 USA

**Keywords:** Illumina sequencing, Oxford Nanopore sequencing, Hybrid assembly, MaSuRCA, SPAdes, Unicycler, Bacterial pathogen, Genomic analyses

## Abstract

**Background:**

We benchmarked the hybrid assembly approaches of MaSuRCA, SPAdes, and Unicycler for bacterial pathogens using Illumina and Oxford Nanopore sequencing by determining genome completeness and accuracy, antimicrobial resistance (AMR), virulence potential, multilocus sequence typing (MLST), phylogeny, and pan genome. Ten bacterial species (10 strains) were tested for simulated reads of both mediocre- and low-quality, whereas 11 bacterial species (12 strains) were tested for real reads.

**Results:**

Unicycler performed the best for achieving contiguous genomes, closely followed by MaSuRCA, while all SPAdes assemblies were incomplete. MaSuRCA was less tolerant of low-quality long reads than SPAdes and Unicycler. The hybrid assemblies of five antimicrobial-resistant strains with simulated reads provided consistent AMR genotypes with the reference genomes. The MaSuRCA assembly of *Staphylococcus aureus* with real reads contained *msr(A)* and *tet(K)*, while the reference genome and SPAdes and Unicycler assemblies harbored *blaZ*. The AMR genotypes of the reference genomes and hybrid assemblies were consistent for the other five antimicrobial-resistant strains with real reads. The numbers of virulence genes in all hybrid assemblies were similar to those of the reference genomes, irrespective of simulated or real reads. Only one exception existed that the reference genome and hybrid assemblies of *Pseudomonas aeruginosa* with mediocre-quality long reads carried 241 virulence genes, whereas 184 virulence genes were identified in the hybrid assemblies of low-quality long reads. The MaSuRCA assemblies of *Escherichia coli* O157:H7 and *Salmonella* Typhimurium with mediocre-quality long reads contained 126 and 118 virulence genes, respectively, while 110 and 107 virulence genes were detected in their MaSuRCA assemblies of low-quality long reads, respectively. All approaches performed well in our MLST and phylogenetic analyses. The pan genomes of the hybrid assemblies of *S.* Typhimurium with mediocre-quality long reads were similar to that of the reference genome, while SPAdes and Unicycler were more tolerant of low-quality long reads than MaSuRCA for the pan-genome analysis. All approaches functioned well in the pan-genome analysis of *Campylobacter jejuni* with real reads.

**Conclusions:**

Our research demonstrates the hybrid assembly pipeline of Unicycler as a superior approach for genomic analyses of bacterial pathogens using Illumina and Oxford Nanopore sequencing.

## Background

Next-generation sequencing (NGS) technologies such as Illumina short-read sequencing have revolutionized whole-genome sequencing (WGS) of bacterial pathogens by increasing throughput exponentially over first-generation Sanger sequencing [1]. Additionally, the use of Illumina sequencing that generates reads with lengths ranging from 50 to 300 bp has improved the accuracy of genomes [2]. Despite the great gains, Illumina sequencing produces highly fragmented assemblies [3], which reveals its limitation in unambiguously resolving long repeats present in multiple copies and GC-rich regions. Illumina short reads make genome assembly difficult as theoretical modeling indicates that reducing read lengths from 1000 bp to 100 bp can cause a six-fold or more decrease in contiguity [4]. Genome fragmentation prevents the accurate identification of mobile genetic elements, detection of horizontal gene transfers, and discovery of microbiologically important genes [5], which significantly lowers the informational value of draft-quality genomes.

The need for sequencing technologies that produce longer reads to overcome the limitations of Illumina sequencing and facilitate the assembly of complete bacterial genomes has resulted in the advent of third-generation sequencing technologies [5]. Oxford Nanopore sequencing is a more recently developed long-read, single-molecule sequencing technology [6], whose read lengths have no theoretical upper limit and can reach up to > 2 million nucleotides [7]. Oxford Nanopore sequencing can generate long reads that span repetitive regions in bacterial genomes, thus resulting in less fragmented or even complete genomes. However, the high error rates of Oxford Nanopore sequencing set a challenge for accurate genomic analyses.

To overcome the limitations of both Illumina and Oxford Nanopore sequencing, as well as unlocking their full potential for genome assembly, a hybrid assembly strategy has been developed. Here, Oxford Nanopore long reads can scaffold contigs generated by Illumina short reads to disambiguate regions of the assembly graph that cannot be resolved by Illumina short reads alone, as implemented in assemblers such as MaSuRCA [8], SPAdes [9], and Unicycler [10].

MaSuRCA combines the benefits of de Bruijn graph and overlap-layout-consensus (OLC) assembly approaches [10]. It supports hybrid assembly with Illumina short reads and Oxford Nanopore long reads [8], which utilizes a submodule of Flye for the final assembly of corrected mega-reads [11, 12]. SPAdes is a genome assembler that was designed for both single-cell and multi-cell bacterial datasets [13]. The current version of SPAdes is capable of providing hybrid assembly (hybridSPAdes) using Oxford Nanopore long reads for gap closure and repeat resolution [14]. SPAdes constructs the de Bruijn assembly graph of *k*-mers from Illumina short reads, maps Oxford Nanopore long reads to the graph to close gaps using the consensus of long reads, and finally resolves repeats by incorporating long-read paths into the decision rule of ExSPAnder. Unicycler is an assembly tool specifically designed for bacterial genomes [10]. The hybrid assembly pipeline of Unicycler produces an Illumina short-read assembly graph and then uses Oxford Nanopore long reads to build bridges, which often allows it to resolve all repeats in the genome and produce a complete genome assembly.

The performance of the hybrid assembly approaches of MaSuRCA, SPAdes, and Unicycler in downstream genomic analyses of bacterial pathogens remains poorly evaluated. In this work, we thus benchmarked the standalone hybrid assembly pipelines of MaSuRCA, SPAdes, and Unicycler for Illumina and Oxford Nanopore sequencing of bacterial pathogens that covered a wide range of genome sizes and GC contents. We assessed each hybrid assembly approach on its ability to generate complete and accurate assemblies for genomic analyses using both simulated and real reads. Simulated reads offer some advantages over real reads when assessing assemblers, which allow for a confident ground truth as the underlying genome is well-established and known with certainty. Meanwhile, by using a long-read simulator such as Badread [15], the quality of simulated reads can be artificially controlled to approximate Oxford Nanopore long reads of differing quality. Despite the advantages of simulated reads, they may sometimes be unrealistic because simulators are not able to model all relevant features of Oxford Nanopore long reads such as error profiles, read lengths, and quality scores. Real reads are therefore also valuable when assessing hybrid assembly approaches. Accordingly, both simulated and real reads were tested in the present study.

## Results and discussion

### Genome completeness and accuracy

Simulated Illumina short reads and Oxford Nanopore long reads (both mediocre and low quality) of each strain were assembled using the hybrid assembly approaches of MaSuRCA, SPAdes, and Unicycler (Tables 1 and 2). The hybrid assemblies were compared to their corresponding ‘gold-standard’ reference genomes. Regarding the simulated long reads that contained artificial error profiles, genome completeness and accuracy reflects the robustness of an assembler to tolerate a variety of read parameters [16]. For mediocre-quality long reads, none of the hybrid assembly approaches managed to produce a complete genome (Table 1). SPAdes was the least robust in terms of contiguity compared to MaSuRCA and Unicycler. Unicycler had the most robust hybrid assembly strategy, closely followed by MaSuRCA. Unicycler produced hybrid assemblies which had the same numbers of contigs as the reference genomes, with only one exception of *S.* Typhimurium LT2, although no contigs were circularized using Unicycler. Only one contig of *C. sakazakii* ATCC 29544 was circularized by MaSuRCA and Unicycler, while none of the contigs were circularized for the other nine strains. MaSuRCA, SPAdes, and Unicycler performed well in terms of genome size and GC content as they all generated accurate genome sizes and GC contents that were similar (*P* > 0.05) to those of the reference genomes. All hybrid assemblies of mediocre-quality long reads had the same (*P* > 0.05) averages of complete (98.4%), fragmented (0.2%), and missing benchmarking universal single-copy orthologs (BUSCOs) (1.4%) (Additional file: Table S9), which were consistent with those of the reference genomes. Lower averages of the numbers of single nucleotide polymorphisms (SNPs) per 1 million bp of the reference genome were identified in the MaSuRCA (0.48) and Unicycler assemblies of mediocre-quality long reads (0.69) than the SPAdes assemblies of mediocre-quality long reads (1.76) (Additional file: Table S12). The numbers of SNPs per 1 million bp of the reference genome of the Unicycler assemblies were significantly lower (*P* < 0.05) than those of the SPAdes assemblies but similar (*P* > 0.05) to those of the MaSuRCA assemblies. No significant differences (*P* > 0.05) were observed between the MaSuRCA and SPAdes assemblies. The MaSuRCA, SPAdes, and Unicycler assemblies of mediocre-quality long reads had similar (*P* > 0.05) averages of OrthoANIu values, which were 99.98, 99.97, and 99.98%, respectively (Additional file: Table S15).

**Table 1:** Hybrid assemblies of bacterial strains with simulated Illumina short reads and mediocre-quality Oxford Nanopore long reads using MaSuRCA, SPAdes, and Unicycler compared to their corresponding reference genomes

Strain	Number of contigs				Total length (bp)				GC content (%)			
	MaSuRCA	SPAdes	Unicycler	Reference	MaSuRCA	SPAdes	Unicycler	Reference	MaSuRCA	SPAdes	Unicycler	Reference
*Pseudomonas aeruginosa* PAO1	1 (0 cir.; 2 dead)	39	1 (0 cir.; 2 dead)	1	6,264,321	6,261,361	6,264,377	6,264,404	66.56	66.57	66.56	66.56
*Escherichia coli* O157:H7 Sakai	2 (0 cir.; 4 dead)	456	3 (0 cir.; 6 dead)	3	5,591,228	5,518,282	5,594,148	5,594,605	50.49	50.40	50.48	50.48
*Bacillus anthracis* Ames Ancestor	3 (0 cir.; 6 dead)	47	3 (0 cir.; 6 dead)	3	5,503,594	5,508,786	5,489,222	5,503,926	35.24	35.25	35.23	35.24
*Klebsiella variicola* DSM 15968	1 (0 cir.; 2 dead)	38	1 (0 cir.; 2 dead)	1	5,521,107	5,517,301	5,521,041	5,521,203	57.56	57.57	57.56	57.56
*Salmonella* Typhimurium LT2	2 (0 cir.; 4 dead)	43	3 (0 cir.; 3 dead)	2	4,951,226	4,956,436	4,947,278	4,951,383	52.24	52.24	52.24	52.24
*Cronobacter sakazakii* ATCC 29544	3 (1 cir.; 4 dead)	54	4 (1 cir.; 6 dead)	4	4,658,297	4,642,775	4,663,118	4,663,565	56.64	56.67	56.64	56.64
*Clostridium botulinum* CDC_1632	2 (1 cir.; 2 dead)	86	1 (0 cir.; 2 dead)	1	4,398,971	4,393,287	4,392,893	4,393,047	28.04	28.01	28.02	28.02
*Listeria monocytogenes* EGD-e	2 (1 cir.; 2 dead)	7	1 (0 cir.; 2 dead)	1	2,950,445	2,944,768	2,944,366	2,944,528	38.01	37.98	37.98	37.98
*Staphylococcus aureus* NCTC 8325	1 (0 cir.; 2 dead)	47	1 (0 cir.; 2 dead)	1	2,821,292	2,825,487	2,821,211	2,821,361	32.87	32.87	32.87	32.87
*Campylobacter jejuni* NCTC 11168	1 (0 cir.; 2 dead)	2	1 (0 cir.; 2 dead)	1	1,641,372	1,641,539	1,641,262	1,641,481	30.55	30.55	30.55	30.55

^a^cir., circularized contigs

^b^dead, dead ends

**Table 2:** Hybrid assemblies of bacterial strains with simulated Illumina short reads and low-quality Oxford Nanopore long reads using MaSuRCA, SPAdes, and Unicycler

Strain	Number of contigs			Total length (bp)			GC content (%)		
	MaSuRCA	SPAdes	Unicycler	MaSuRCA	SPAdes	Unicycler	MaSuRCA	SPAdes	Unicycler
*Pseudomonas aeruginosa* PAO1	4 (0 cir.; 8 dead)	64	1 (0 cir.; 2 dead)	6,173,430	6,261,368	6,264,384	66.55	66.57	66.56
*Escherichia coli* O157:H7 Sakai	6 (0 cir.; 12 dead)	550	83 (0 cir.; 6 dead)	5,503,884	5,487,649	5,562,446	50.50	50.38	50.45
*Bacillus anthracis* Ames Ancestor	7 (0 cir.; 14 dead)	78	3 (0 cir.; 6 dead)	5,433,227	5,482,457	5,489,222	35.26	35.17	35.23
*Klebsiella variicola* DSM 15968	1 (0 cir.; 2 dead)	121	1 (0 cir.; 2 dead)	5,482,918	5,505,259	5,520,752	57.54	57.57	57.56
*Salmonella* Typhimurium LT2	3 (0 cir.; 6 dead)	78	3 (0 cir.; 3 dead)	4,848,561	4,952,963	4,947,288	52.22	52.24	52.24
*Cronobacter sakazakii* ATCC 29544	18 (0 cir.; 36 dead)	72	4 (1 cir.; 6 dead)	4,492,402	4,640,822	4,663,144	56.60	56.67	56.64
*Clostridium botulinum* CDC_1632	2 (1 cir.; 2 dead)	109	11 (0 cir.; 2 dead)	4,398,971	4,379,115	4,372,396	28.04	27.95	27.92
*Listeria monocytogenes* EGD-e	4 (1 cir.; 6 dead)	11	1 (0 cir.; 2 dead)	2,927,219	2,933,282	2,942,862	38.02	37.94	37.97
*Staphylococcus aureus* NCTC 8325	3 (0 cir.; 6 dead)	37	1 (0 cir.; 2 dead)	2,759,087	2,818,488	2,821,119	32.88	32.84	32.87
*Campylobacter jejuni* NCTC 11168	1 (0 cir.; 2 dead)	8	1 (0 cir.; 2 dead)	1,630,638	1,640,924	1,641,330	30.56	30.55	30.55

^a^cir., circularized contigs

^b^dead, dead ends

We also used simulated Oxford Nanopore long reads of low quality to examine if the hybrid assembly approaches of MaSuRCA, SPAdes, and Unicycler could tolerate more sequence errors (Table 2). Similar to mediocre-quality long reads, neither MaSuRCA, SPAdes, nor Unicycler managed to complete the genomes using low-quality long reads. However, all hybrid assembly approaches produced more fragmented contigs using low-quality long reads than mediocre-quality long reads. Unicycler was the most robust to long-read quality, as inferred by the similarity in the number of contigs produced using mediocre- and low-quality long reads, although the Unicycler assemblies of *E. coli* O157:H7 Sakai and *Clostridium botulinum* CDC_1632 with low-quality long reads produced much more highly fragmented assemblies, with 83 and 11 contigs, respectively. Relatively smaller genome sizes were found compared to mediocre-quality long reads, especially for the MaSuRCA assemblies. The hybrid assemblies had significantly smaller (*P* < 0.05) genome sizes than the reference genomes, although the genome sizes of the SPAdes and Unicycler assemblies were significantly higher (*P* < 0.05) than those of the MaSuRCA assemblies. There were no significant differences (*P* > 0.05) in genome size between the SPAdes and Unicycler assemblies. The hybrid assemblies of low-quality long reads had similar GC contents to those of mediocre-quality long reads. There were no significant differences (*P* > 0.05) in GC content among the reference genomes, MaSuRCA, SPAdes, and Unicycler assemblies of low-quality long reads. Compared to the MaSuRCA assemblies of mediocre-quality long reads, a noticeable decrease in the average of complete BUSCOs (94.5%) was observed for those of low-quality long reads (Additional file: Table S10). There were increases in the averages of fragmented (2.1%) and missing BUSCOs (3.4%) of the MaSuRCA assemblies of low-quality long reads compared to those of mediocre-quality long reads. In contrast, the BUSCO profiles in the SPAdes and Unicycler assemblies of low-quality long reads remained the same as those of mediocre-quality long reads. The complete BUSCOs of the MaSuRCA assemblies of low-quality long reads were significantly lower (*P* < 0.05) than those of the reference genomes, while their fragmented and missing BUSCOs were significantly higher (*P* < 0.05) than those of the reference genomes. No significant differences (*P* > 0.05) in complete, fragmented, and missing BUSCOs were found among the reference genomes, the SPAdes and Unicycler assemblies. Interestingly, compared to mediocre-quality long reads, even lower averages of the numbers of SNPs per one million bp of the reference genome were observed in the SPAdes (1.45) and Unicycler assemblies (0.32) of low-quality long reads (Additional file: Table S13), with no significant differences (*P* > 0.05) between them, while the MaSuRCA assemblies had a significantly higher (*P* < 0.05) average of the numbers of SNPs per 1 million bp of the reference genome (2.54). The SPAdes and Unicycler assemblies of low-quality long reads had similar averages of OrthoANIu values than those of mediocre-quality long reads, which were 99.96 and 99.98%, respectively (Additional file: Table S16). No significant differences (*P* > 0.05) in OrthoANIu value were found between the SPAdes and Unicycler assemblies. However, we found that the MaSuRCA assemblies of low-quality long reads had a lower average of OrthoANIu value (99.82%) than that of mediocre-quality long reads, suggesting that the differences between the reference genomes and MaSuRCA assemblies became greater when more long-read errors were introduced. The OrthoANIu values of the MaSuRCA assemblies were significantly lower (*P* < 0.05) than those of the SPAdes and Unicycler assemblies.

The genome completeness and accuracy of an assembly given a set of real reads indicates the reliability to achieve a complete and accurate assembly, which incorporate naturally occurring features of Oxford Nanopore long reads [16]. The MaSuRCA and Unicycler assemblies of real reads were more contiguous than those of simulated reads, while SPAdes failed to complete any of the genomes (Table 3). Similarly, Golparian et al. [17] reported that the SPAdes assemblies of *Neisseria gonorrhoeae* strains with real reads had less contiguity than the MaSuRCA assemblies. Goldstein et al. [18] also observed that Unicycler consistently outperformed SPAdes in terms of contiguity during hybrid assembly of *Flavobacterium*, *Aeromonas*, and *Pseudonocardia* strains with real reads. In our study, Unicycler performed the best and completed the genomes of 10 strains, with only two exceptions of *E. coli* O26:H11 CFSAN027343 and *E. coli* O26:H11 CFSAN027350. Among the 10 genomes Unicycler completed, only the Unicycler assembly of *Staphylococcus aureus* CFSAN007894 had inconsistent numbers of contigs (three contigs) compared to the reference genome (two contigs). MaSuRCA failed to complete the genomes of five strains but produced assemblies of the seven other strains that had consistent numbers of contigs with the reference genomes. There were no significant differences (*P* > 0.05) in genome size and GC content among the reference genomes, MaSuRCA, SPAdes, and Unicycler assemblies of real reads. The MaSuRCA assemblies had averages of complete and missing BUSCOs of 97.1 and 2.4%, respectively (Additional file: Table S11), while the averages of complete and missing BUSCOs of the SPAdes and Unicycler assemblies were 97.3 and 2.3%, respectively, which were congruent with those of the reference genome. The reference genomes and hybrid assemblies had the same average of fragmented BUSCOs (0.5%). The complete, fragmented, and missing BUSCOs of the reference genomes, MaSuRCA, SPAdes, and Unicycler assemblies of real reads were similar (*P* > 0.05). The Unicycler assemblies had a significantly lower (*P* < 0.05) average of the numbers of SNPs per 1 million bp of the reference genome (0.57) than the MaSuRCA (3.99) assemblies (Additional file: Table S14), while there were no significant differences (*P* > 0.05) between the MaSuRCA and SPAdes (0.82) assemblies. The averages of the numbers of SNPs per 1 million bp of the reference genome of the SPAdes and Unicycler assemblies were also similar (*P* > 0.05). Similar (*P* > 0.05) averages of OrthoANIu values were observed for the MaSuRCA, SPAdes, and Unicycler assemblies, which were 99.97, 99.98, and 99.97%, respectively (Additional file: Table S17).

**Table 3:** Hybrid assemblies of bacterial strains with real Illumina short reads and Oxford Nanopore long reads using MaSuRCA, SPAdes, and Unicycler compared to their corresponding reference genomes

Strain	Number of contigs				Total length (bp)				GC content (%)			
	MaSuRCA	SPAdes	Unicycler	Reference	MaSuRCA	SPAdes	Unicycler	Reference	MaSuRCA	SPAdes	Unicycler	Reference
*Escherichia coli* O26:H11 CFSAN027343	2 (2 cir.; 1 dead)	585	97 (3 cir.; 0 dead)	2	5,803,063	5,674,058	5,722,981	5,778,004	50.67	50.57	50.67	50.68
*Escherichia coli* O26:H11 CFSAN027350	5 (0 cir.; 2 dead)	488	108 (1 cir.; 0 dead)	2	5,604,298	5,512,883	5,522,347	5,593,613	50.65	50.53	50.57	50.64
*Klebsiella variicola* CFSAN086180	1 (1 cir.; 0 dead)	89	1 (1 cir.; 0 dead)	1	5,536,665	5,543,412	5,536,929	5,536,659	57.53	57.53	57.53	57.53
*Klebsiella pneumonia* CFSAN086181	1 (1 cir.; 0 dead)	56	1 (1 cir.; 0 dead)	1	5,221,910	5,230,821	5,221,909	5,221,909	57.58	57.53	57.58	57.58
*Enterobacter cancerogenus* CFSAN086183	2 (2 cir.; 0 dead)	113	2 (2 cir.; 0 dead)	2	4,990,691	5,003,034	4,992,376	4,992,376	55.59	55.56	55.59	55.59
*Salmonella* Bareilly CFSAN000189	2 (2 cir.; 0 dead)	80	2 (2 cir.; 0 dead)	2	4,806,601	4,816,325	4,806,603	4,808,805	52.21	52.23	52.21	52.22
*Citrobacter braakii* CFSAN086182	1 (1 cir.; 0 dead)	66	1 (1 cir.; 0 dead)	1	4,917,488	4,939,416	4,917,491	4,917,511	52.05	51.99	52.05	52.05
*Cronobacter sakazakii* CFSAN068773	3 (2 cir.; 1 dead)	75	4 (4 cir.; 0 dead)	4	4,577,802	4,597,231	4,581,781	4,581,781	56.73	56.71	56.73	56.73
*Listeria monocytogenes* CFSAN008100	2 (2 cir.; 1 dead)	18	2 (2 cir.; 0 dead)	2	3,102,056	3,140,938	3,137,527	3,108,102	37.95	37.91	37.90	37.91
*Staphylococcus aureus* CFSAN007894	4 (1 cir.; 6 dead)	75	3 (3 cir.; 0 dead)	2	2,749,203	2,774,416	2,763,803	2,757,659	32.86	32.92	32.82	32.84
*Campylobacter jejuni* CFSAN032806	2 (2 cir.; 0 dead)	55	2 (2 cir.; 0 dead)	2	1,811,805	1,774,178	1,814,450	1,782,911	30.53	30.67	30.54	30.53
*Campylobacter coli* CFSAN032805	3 (3 cir.; 0 dead)	51	3 (3 cir.; 0 dead)	3	1,750,134	1,765,016	1,750,172	1,750,177	31.41	31.56	31.41	31.41

^a^cir., circularized contigs

^b^dead, dead ends

As predicted based on the PlasmidFinder database, hybrid assemblies using different approaches showed consistent plasmid profiles with their corresponding reference genomes, with a few exceptions for both simulated and real reads (Additional files: Tables S18, S19, S20). Compared to the reference genome, the Unicycler assembly of *S.* Typhimurium LT2 with mediocre- and low-quality long reads did not contain IncFII (S), while IncFIB (S) and IncFII (S) were not identified in the MaSuRCA assembly of low-quality long reads. The Unicycler assembly of *E. coli* O26:H11 CFSAN027343 failed to carry IncB/O/K/Z that was detected in the reference genome. Noticeably, according to the Unicycler assembly of *S. aureus* CFSAN007894 with real reads, we successfully assembled a small plasmid (2,491 bp) that was missing in the PacBio-based reference genome. Careful DNA extraction and library preparation are crucial to isolate and sequence the longest molecules possible for long-read sequencing such as PacBio and Oxford Nanopore sequencing. Size selection to enrich long DNA fragments during long-read sequencing library preparation can inadvertently exclude short plasmids [19], so a secondary, short-fragment library to retain shorter DNA fragments may sometimes be required.

Among the ten genomes Unicycler completed, only the Unicycler assembly of *Staphylococcus aureus* CFSAN007894 had inconsistent numbers of contigs (three contigs) compared to the reference genome (two contigs). MaSuRCA failed to complete the genomes of five strains but produced assemblies of the seven other strains that had consistent numbers of contigs with the reference genomes.

According to the assembly results using both simulated and real reads, all hybrid assembly approaches generated assemblies that had consistent genome sizes and GC contents with the reference genomes, whereas the SPAdes assemblies were inferior in contiguity compared to both the MaSuRCA and Unicycler assemblies. MaSuRCA was an outlier of assembling accurate genomes using low-quality long reads compared to SPAdes and Unicycler. Overall, Unicycler emerged as the best hybrid assembly performer that was able to tolerate both simulated and real reads. Wick et al. [9] also demonstrated the advantage of Unicycler over SPAdes in fully resolving the genomic structures of 12 strains of *Klebsiella pneumoniae* during hybrid assemblies.

### Antimicrobial resistance genes (ARGs)

Understanding the genomic environments of ARGs to explore whether they are chromosomally encoded or plasmid-encoded is essential for monitoring the transmission of ARGs and assessing the risk to public health. Illumina short reads can identify the presence or absence of ARGs but not their genomic architectures [20]. Oxford Nanopore sequencing enables the identification of mobile genetic elements on which ARGs are located and also characterizes the combination of different ARGs co-located on the same mobile genetic element. A combination of Illumina short reads and Oxford Nanopore long reads can contribute to a better understanding of the location of ARGs in antimicrobial-resistant bacterial pathogens. The higher error rates of Oxford Nanopore long reads could be compensated for by bioinformatic algorithms through hybrid assembly to acquire more accurate AMR profiling [21]. Abdelhamed et al. [22] closed the complete genome of a multidrug-resistant *Plesiomonas shigelloides* strain, which was assembled with Illumina short reads and Oxford Nanopore long reads using MaSuRCA. As revealed by the MaSuRCA assembly, among the three plasmids identified in this strain, one was found to carry multiple ARGs. To resolve the structure of a composite AMR island in a *S.* Typhi strain, Ashton et al. [23] assembled Illumina short reads and Oxford Nanopore long reads using SPAdes. The SPAdes assembly confirmed the *yidA* insertion site but failed to resolve the structure with breaks between *hyp* and *merA* because the genome was resolved into as many as 34 contigs. To explore the genomic environment of a multidrug-resistant enteroaggregative *E. coli* O51: H30 strain, Greig et al. [20] used Unicycler to obtain the hybrid assembly of Illumina short reads and Oxford Nanopore long reads. They found that the majority of the 12 ARGs identified in this strain clustered together on the chromosome at three separate locations flanked by integrases and/or insertion elements.

In the present study, we compared the genotypes and predicted phenotypes of AMR of bacterial pathogens, as predicted based on the MaSuRCA, SPAdes, and Unicycler assemblies. Five genotypically antimicrobial-resistant strains with simulated reads were used for the benchmarking of hybrid assembly approaches (Table 4). The MaSuRCA, SPAdes, and Unicycler assemblies of mediocre-quality long reads provided consistent genotypes and predicted phenotypes with their corresponding reference genomes, indicating that they were all capable of acquiring hybrid assemblies that can be used for accurate predictions of AMR phenotypes. The MaSuRCA, SPAdes, and Unicycler assemblies of low-quality long reads also performed well, which showed congruent genotypes and predicted phenotypes with those of mediocre-quality long reads. Based on the MaSuRCA and Unicycler assemblies, the only ARG (*fosB2*) in *Bacillus anthracis* Ames Ancestor was located on the chromosome, although its genome contained two plasmids. All of the ARGs in the other four genotypically antimicrobial-resistant strains were also located on the chromosome. Our results show that the SPAdes assemblies were not able to indicate if ARGs were located on the chromosome or plasmid(s) due to incomplete genomes.

**Table 4:** Genotypes and predicted phenotypes of antimicrobial resistance (AMR) of bacterial strains with simulated Illumina short reads and either mediocre-quality or low-quality Oxford Nanopore long reads, as predicted based on their MaSuRCA, SPAdes, and Unicycler assemblies and compared to their corresponding reference genomes^a^

Strain	Genotype				Predicted phenotype			
	MaSuRCA	SPAdes	Unicycler	Reference	MaSuRCA	SPAdes	Unicycler	Reference
*Pseudomonas aeruginosa* PAO1	*aph(3′)-IIb* *blaOXA-50* *blaPAO* *catB7* *fosA*	*aph(3′)-IIb* *blaOXA-50* *blaPAO* *catB7* *fosA*	*aph(3′)-IIb* *blaOXA-50* *blaPAO* *catB7* *fosA*	*aph(3′)-IIb* *blaOXA-50* *blaPAO* *catB7* *fosA*	Kanamycin Ampicillin Amoxicillin/clavulanic acid Cefoxitin Ceftriaxone Chloramphenicol Fosfomycin	Kanamycin Ampicillin Amoxicillin/clavulanic acid Cefoxitin Ceftriaxone Chloramphenicol Fosfomycin	Kanamycin Ampicillin Amoxicillin/clavulanic acid Cefoxitin Ceftriaxone Chloramphenicol Fosfomycin	Kanamycin Ampicillin Amoxicillin/clavulanic acid Cefoxitin Ceftriaxone Chloramphenicol Fosfomycin
*Bacillus anthracis* Ames Ancestor	*fosB2*	*fosB2*	*fosB2*	*fosB2*	Fosfomycin	Fosfomycin	Fosfomycin	Fosfomycin
*Klebsiella variicola* DSM 15968	*blaLEN17*	*blaLEN17*	*blaLEN17*	*blaLEN17*	Ampicillin	Ampicillin	Ampicillin	Ampicillin
*Listeria monocytogenes* EGD-e	*fosX*	*fosX*	*fosX*	*fosX*	Fosfomycin	Fosfomycin	Fosfomycin	Fosfomycin
*Campylobacter jejuni* NCTC 11168	*blaOXA-61*	*blaOXA-61*	*blaOXA-61*	*blaOXA-61*	Ampicillin	Ampicillin	Ampicillin	Ampicillin

^a^No antimicrobial resistance genes (ARGs) were detected in *E. coli* O157:H7 Sakai, *S.* Typhimurium LT2, *C. sakazakii* ATCC 29544, or *S. aureus* NCTC 8325 as predicted based on their hybrid assemblies using MaSuRCA, SPAdes, and Unicycler

There were six genotypically antimicrobial-resistant strains with real reads (Table 5). Noticeably, two ARGs, *msr(A)* and *tet(K)*, were detected in the MaSuRCA assembly of *S. aureus* CFSAN007894, which was inconsistent with the reference genome and SPAdes and Unicycler assemblies that harbored *blaZ*. This was the only case where an inferred AMR phenotype differed among the reference genome and hybrid assemblies. The Unicycler assembly indicates that *blaZ* was located on a plasmid, while the MaSuRCA and SPAdes assemblies could not suggest the locations of ARGs due to incomplete genomes. The hybrid assemblies of the other five antimicrobial-resistant strains had consistent genotypes and predicted phenotypes with their corresponding reference genomes. We found that both *C. jejuni* CFSAN032806 and *C. coli* CFSAN032805 had one ARG (*blaOXA-61*) located on the chromosome and three ARGs [*aph (2″)-Ig*, *aph (3′)-III*, and *tet(O)*] co-located on a single plasmid. Each of the other three genotypically antimicrobial-resistant strains carried ARGs on the chromosome, as predicted based on the MaSuRCA and Unicycler assemblies.

**Table 5:** Genotypes and predicted phenotypes of antimicrobial resistance (AMR) of bacterial strains with real Illumina short reads and Oxford Nanopore long reads, as predicted based on their MaSuRCA, SPAdes, and Unicycler assemblies and compared to their corresponding reference genomes^a^

Strain	Genotype				Predicted phenotype			
	MaSuRCA	SPAdes	Unicycler	Reference	MaSuRCA	SPAdes	Unicycler	Reference
*Klebsiella variicola* CFSAN086180	*blaLEN16*	*blaLEN16*	*blaLEN16*	*blaLEN16*	Ampicillin	Ampicillin	Ampicillin	Ampicillin
*Klebsiella pneumonia* CFSAN086181	*blaSHV-11* *fosA6* *oqxA* *oqxB*	*blaSHV-11* *fosA6* *oqxA* *oqxB*	*blaSHV-11* *fosA6* *oqxA* *oqxB*	*blaSHV-11* *fosA6* *oqxA* *oqxB*	Ampicillin Fosfomycin Chloramphenicol	Ampicillin Fosfomycin Chloramphenicol	Ampicillin Fosfomycin Chloramphenicol	Ampicillin Fosfomycin Chloramphenicol
*Citrobacter braakii* CFSAN086182	*blaCMY-83*	*blaCMY-83*	*blaCMY-83*	*blaCMY-83*	Ampicillin Amoxicillin/clavulanic acid Cefoxitin Ceftriaxone	Ampicillin Amoxicillin/clavulanic acid Cefoxitin Ceftriaxone	Ampicillin Amoxicillin/clavulanic acid Cefoxitin Ceftriaxone	Ampicillin Amoxicillin/clavulanic acid Cefoxitin Ceftriaxone
*Staphylococcus aureus* CFSAN007894	*msr(A)* *tet(K)*	*blaZ*	*blaZ*	*blaZ*	Erythromycin Azithromycin Tetracycline	Ampicillin	Ampicillin	Ampicillin
*Campylobacter jejuni* CFSAN032806	*aph(2″)-Ig* *aph(3′)-III* *blaOXA-61* *tet(O)*	*aph(2″)-Ig* *aph(3′)-III* *blaOXA-61* *tet(O)*	*aph(2″)-Ig* *aph(3′)-III* *blaOXA-61* *tet(O)*	*aph(2″)-Ig* *aph(3′)-III* *blaOXA-61* *tet(O)*	Kanamycin Ampicillin Tetracycline	Kanamycin Ampicillin Tetracycline	Kanamycin Ampicillin Tetracycline	Kanamycin Ampicillin Tetracycline
*Campylobacter coli* CFSAN032805	*aph(2″)-Ig* *aph(3′)-III* *blaOXA-61* *tet(O)*	*aph(2″)-Ig* *aph(3′)-III* *blaOXA-61* *tet(O)*	*aph(2″)-Ig* *aph(3′)-III* *blaOXA-61* *tet(O)*	*aph(2″)-Ig* *aph(3′)-III* *blaOXA-61* *tet(O)*	Kanamycin Ampicillin Tetracycline	Kanamycin Ampicillin Tetracycline	Kanamycin Ampicillin Tetracycline	Kanamycin Ampicillin Tetracycline

^a^No antimicrobial resistance genes (ARGs) were detected in *E. coli* O26:H11 CFSAN027343, *E. coli* O26:H11 CFSAN027350, *E. cancerogenus* CFSAN086183, *S.* Bareilly CFSAN000189, *C. sakazakii* CFSAN068773, or *L. monocytogenes* CFSAN008100, as predicted based on their hybrid assemblies using MaSuRCA, SPAdes, and Unicycler

The aim of performing AMR predictions based solely on genomic information of bacterial pathogens demands both complete and accurate genomes. Oxford Nanopore sequencing that provides real-time sequencing will also help implement near real-time AMR profiling. While it is feasible to assemble Oxford Nanopore long reads alone into complete genomes [24], doing so would compromise the genome accuracy of bacterial pathogens, which could lead to incorrect AMR profiling [21]. Future improvements to library preparation, basecalling, and long-read-only assembly algorithms may mitigate this limitation, but until then both Illumina short reads and Oxford Nanopore long reads are needed to produce best assemblies of bacterial pathogens, as demonstrated in our study.

### Virulence genes

Although WGS provides detailed information that will theoretically enable routine virulence profiling of bacterial pathogens, it is a challenge to extract the most appropriate information from a large amount of sequence data. Thus, to facilitate the use of WGS data for outbreak surveillance and investigations, the sequence data must be accurately assembled to include all relevant virulence genes. Turton et al. [25] revealed the virulence profile of a virulence plasmid (pKpvST147L) in a *K. pneumoniae* strain using the SPAdes assembly of Illumina short reads and Oxford Nanopore long reads. Ruan et al. [26] identified a set of virulence genes on an IncFIB/IncHI1B plasmid in a *K. pneumoniae* strain based on the Unicycler assembly of Illumina short reads and Oxford Nanopore long reads. To our knowledge, the use of the MaSuRCA assemblies of Illumina short reads and Oxford Nanopore long reads to identify virulence genes of bacterial pathogens has not been reported.

As compared to the reference genomes, the numbers of virulence genes in the MaSuRCA, SPAdes, and Unicycler assemblies of simulated reads were not significantly different (*P* > 0.05), whereas the numbers of virulence genes in the MaSuRCA, SPAdes, and Unicycler assemblies of real reads were significantly lower (*P* < 0.05). We found that the numbers of virulence genes identified in the MaSuRCA, SPAdes, and Unicycler assemblies of each strain were similar (*P* > 0.05), irrespective of simulated or real reads (Tables 6, 7, 8). Concerning the identification of virulence genes, all hybrid assembly approaches could tolerate a higher level of error in low-quality long reads. There was one notable exception that the hybrid assemblies of *P. aeruginosa* PAO1 with mediocre-quality long reads carried up to 241 virulence genes, which were consistent with the reference genome, whereas only 184 virulence genes were present in the hybrid assemblies of low-quality long reads. The MaSuRCA assemblies of *E. coli* O157:H7 Sakai and *S.* Typhimurium LT2 with mediocre-quality long reads harbored 126 and 118 virulence genes, respectively, which were consistent with their corresponding reference genomes, while only 110 and 107 virulence genes were detected in the MaSuRCA assemblies of low-quality long reads, respectively.

**Table 6:** Numbers of virulence genes of bacterial strains with simulated Illumina short reads and mediocre-quality Oxford Nanopore long reads, as predicted based on their MaSuRCA, SPAdes, and Unicycler assemblies and compared to their corresponding reference genomes

Strain	Number of virulence genes			
	MaSuRCA	SPAdes	Unicycler	Reference
*Pseudomonas aeruginosa* PAO1	241	241	241	241
*Escherichia coli* O157:H7 Sakai	126	128	126	126
*Bacillus anthracis* Ames Ancestor	13	13	13	13
*Klebsiella variicola* DSM 15968	10	10	10	10
*Salmonella* Typhimurium LT2	118	118	118	118
*Cronobacter sakazakii* ATCC 29544	2	2	2	2
*Clostridium botulinum* CDC_1632	0	0	0	0
*Listeria monocytogenes* EGD-e	32	32	32	32
*Staphylococcus aureus* NCTC 8325	63	63	63	63
*Campylobacter jejuni* NCTC 11168	118	119	119	119

**Table 7:** Numbers of virulence genes of bacterial strains with simulated Illumina short reads and low-quality Oxford Nanopore long reads, as predicted based on their MaSuRCA, SPAdes, and Unicycler assemblies

Strain	Number of virulence genes		
	MaSuRCA	SPAdes	Unicycler
*Pseudomonas aeruginosa* PAO1	184	184	184
*Escherichia coli* O157:H7 Sakai	110	128	128
*Bacillus anthracis* Ames Ancestor	13	13	13
*Klebsiella variicola* DSM 15968	10	10	10
*Salmonella* Typhimurium LT2	107	117	118
*Cronobacter sakazakii* ATCC 29544	2	2	2
*Clostridium botulinum* CDC_1632	0	0	0
*Listeria monocytogenes* EGD-e	32	32	32
*Staphylococcus aureus* NCTC 8325	62	63	63
*Campylobacter jejuni* NCTC 11168	118	119	118

**Table 8:** Numbers of virulence genes of bacterial strains with real Illumina short reads and Oxford Nanopore long reads, as predicted based on their MaSuRCA, SPAdes, and Unicycler assemblies and compared to their corresponding reference genomes

Strain	Numbers of virulence genes			
	MaSuRCA	SPAdes	Unicycler	Reference
*Escherichia coli* O26:H11 CFSAN027343	115	115	114	121
*Escherichia coli* O26:H11 CFSAN027350	110	108	109	115
*Klebsiella variicola* CFSAN086180	10	10	10	10
*Klebsiella pneumonia* CFSAN086181	10	10	10	10
*Enterobacter cancerogenus* CFSAN086183	15	15	15	16
*Salmonella* Bareilly CFSAN000189	109	109	109	111
*Citrobacter braakii* CFSAN086182	23	23	23	22
*Cronobacter sakazakii* CFSAN068773	2	2	2	4
*Listeria monocytogenes* CFSAN008100	31	31	31	32
*Staphylococcus aureus* CFSAN007894	63	63	63	63
*Campylobacter jejuni* CFSAN032806	104	105	104	107
*Campylobacter coli* CFSAN032805	76	76	76	77

### Multilocus sequence typing (MLST)

Traditional molecular typing schemes for the characterization of bacterial pathogens are poorly portable due to the index variation that is difficult to compare among laboratories. To overcome this deficiency, MLST was proposed by Maiden et al. [27] to exploit the unambiguous nature and electronic portability of nucleotide sequence data of bacterial pathogens. However, MLST is traditionally performed in an expensive and time-consuming manner. As the costs of WGS continues to decline, WGS data of an increasing number of bacterial pathogens are now becoming publicly available. It is recommended that an important consideration of an accurate MLST based on WGS data should be the quality of genome assemblies [28], which is generally controlled at the assembly stage. Accordingly, the new challenge will be how to achieve high-quality assemblies from a large amount of WGS data to allow for an accurate MLST.

In this study, we investigated if MaSuRCA, SPAdes, and Unicycler could be used to produce hybrid assemblies of bacterial pathogens for an accurate MLST. We observed highly consistent positive results of MLST among the MaSuRCA, SPAdes, and Unicycler assemblies of each strain, irrespective of whether they were generated using simulated or real reads. Therefore, the hybrid assembly approaches of MaSuRCA, SPAdes, and Unicycler enabled an accurate MLST based on Illumina short reads and Oxford Nanopore long reads, even in the case where low-quality long reads were used.

### Whole- and core-genome phylogeny

**Fig. 1:**
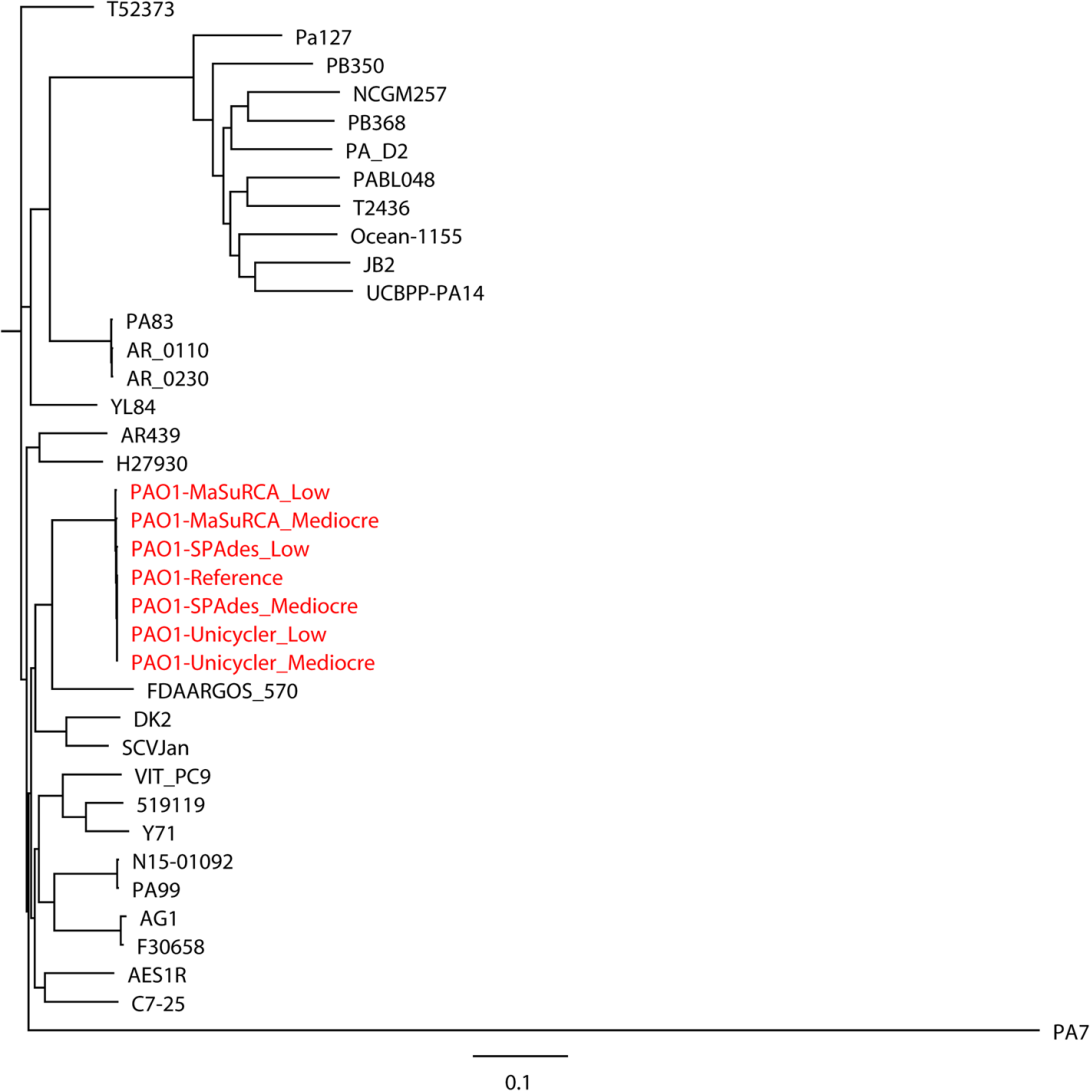
Whole-genome phylogenetic tree of the hybrid assemblies of *Pseudomonas aeruginosa* PAO1 with simulated Illumina short reads and mediocre- or low-quality Oxford Nanopore long reads using MaSuRCA, SPAdes, and Unicycler in addition to the reference genome (in red) compared to 30 *P. aeruginosa* strains. The scale bar indicates the genetic distance

**Fig. 2:**
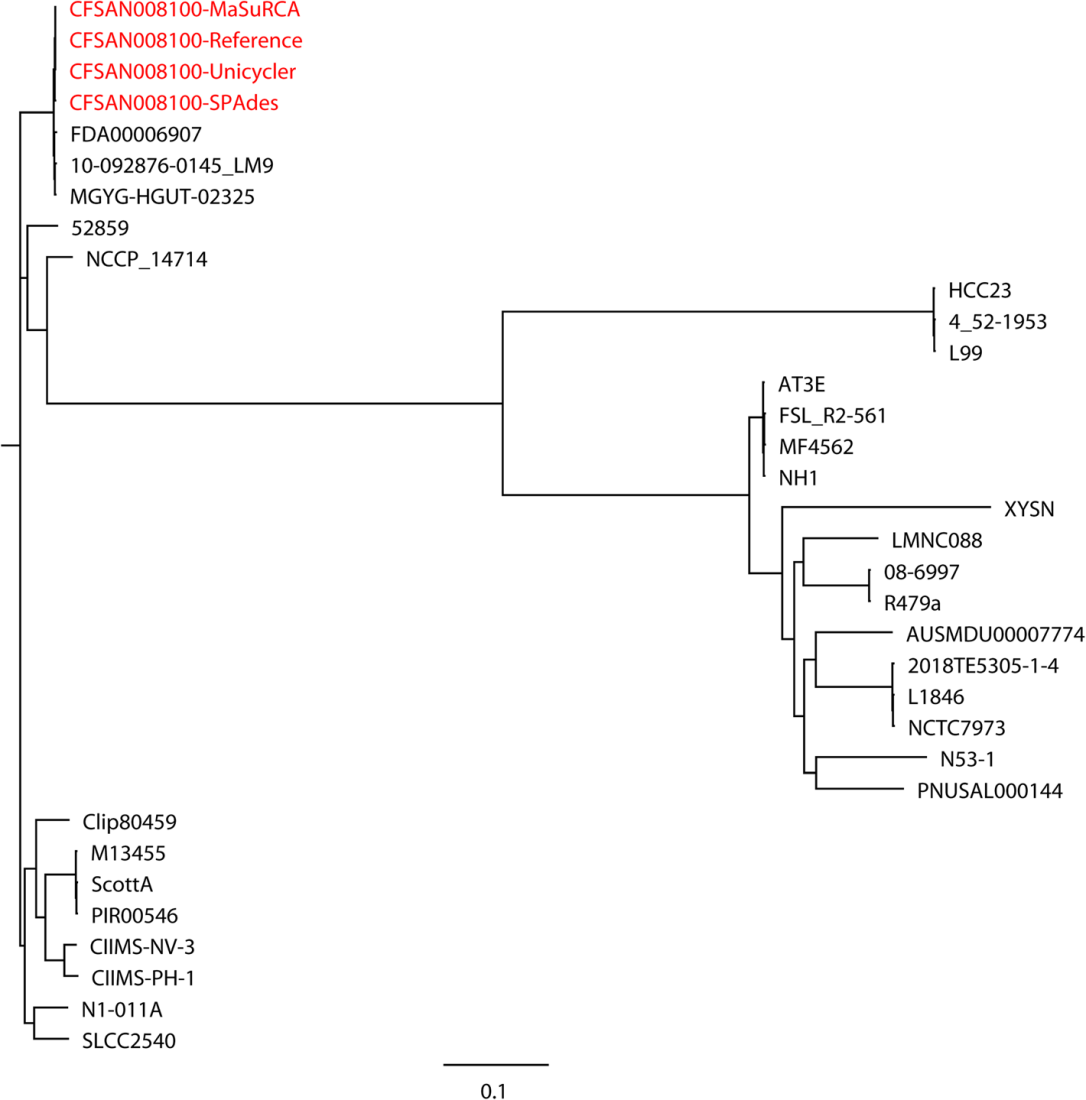
Whole-genome phylogenetic tree of the hybrid assemblies of *Listeria monocytogenes* CFSAN008100 with real Illumina short reads and Oxford Nanopore long reads using MaSuRCA, SPAdes, and Unicycler in addition to the reference genome (in red) compared to 30 *L. monocytogenes* strains. The scale bar indicates the genetic distance

**Fig. 3:**
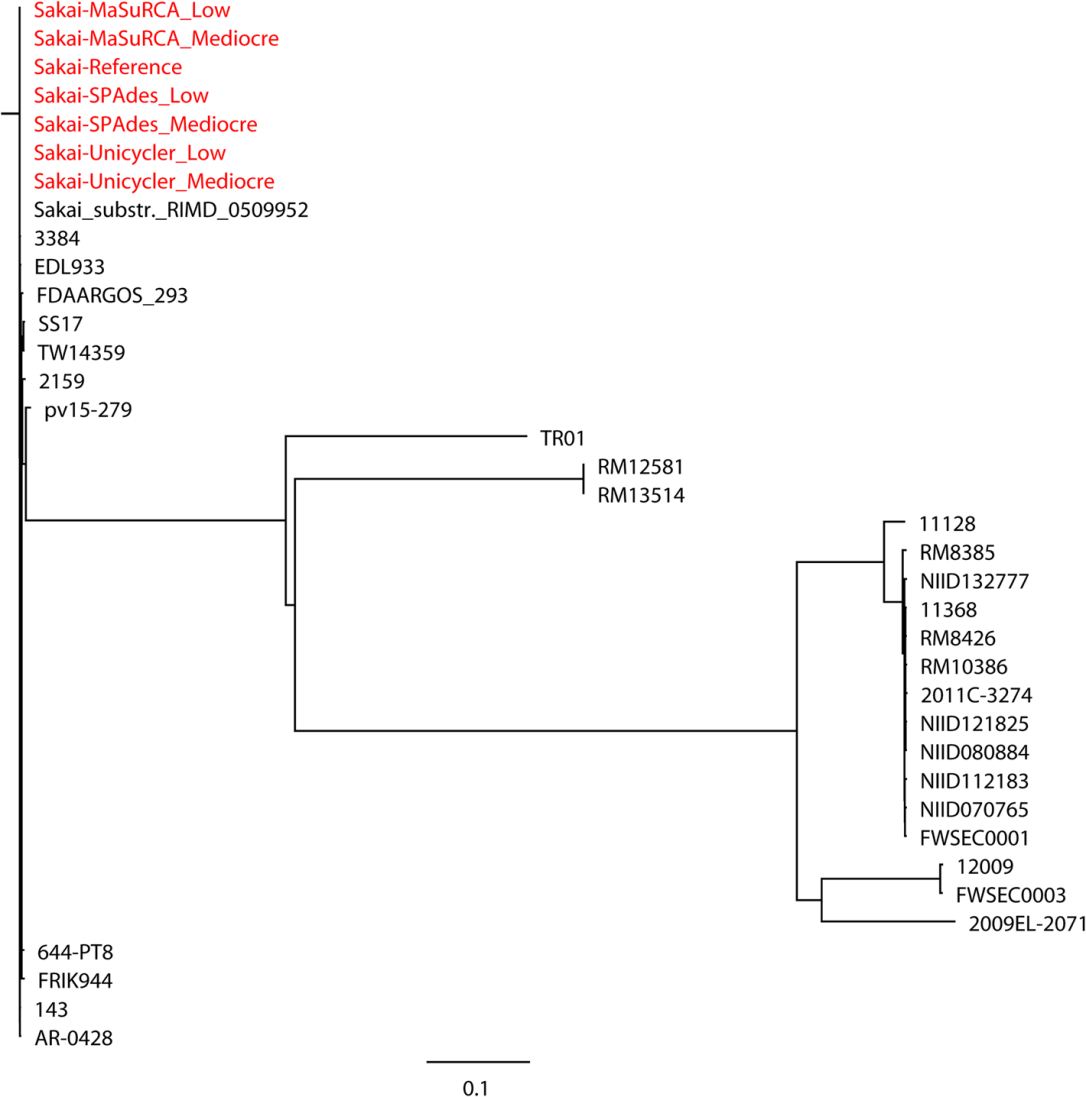
Core-genome phylogenetic tree of the hybrid assemblies of *Escherichia coli* O157:H7 Sakai with simulated Illumina short reads and mediocre- or low-quality Oxford Nanopore long reads using MaSuRCA, SPAdes, and Unicycler in addition to the reference genome (in red) compared to 30 Shiga-toxin producing *E. coli* (STEC) strains. The scale bar indicates the genetic distance

**Fig. 4:**
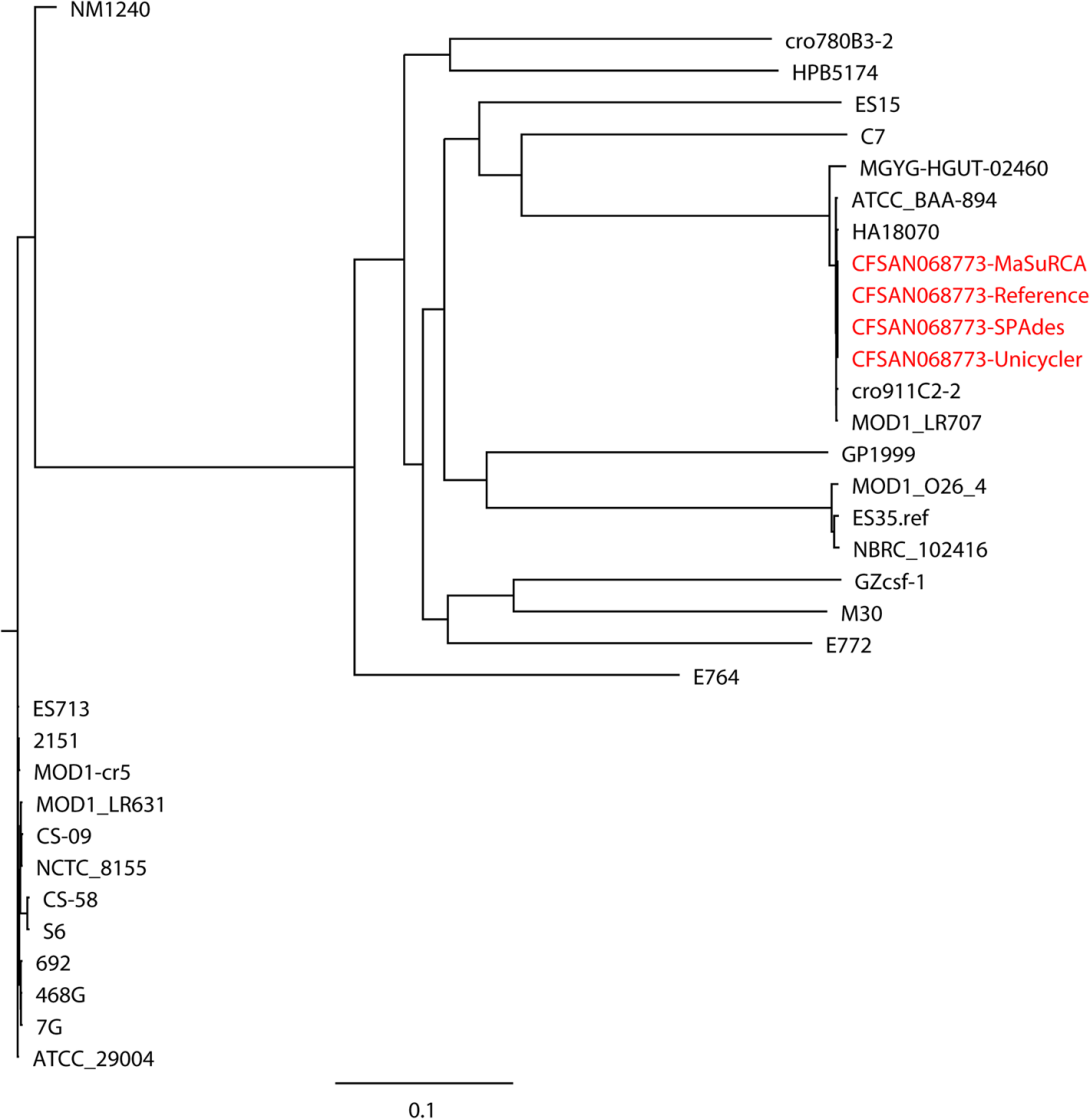
Core-genome phylogenetic tree of the hybrid assemblies of *Cronobacter sakazakii* CFSAN068773 with real Illumina short reads and Oxford Nanopore long reads using MaSuRCA, SPAdes, and Unicycler in addition to the reference genome (in red) compared to 30 *C. sakazakii* strains. The scale bar indicates the genetic distance

The hybrid assemblies of MaSuRCA, SPAdes, and Unicycler have previously been used to build phylogenetic trees of bacterial pathogens such as intestinal pathogenic *E. coli* (IPEC), extraintestinal pathogenic *E. coli* (ExPEC), *N. gonorrhoeae*, *S.* Goldcoast, and *K. pneumoniae* [17, 26, 29, 30]. In the current study, we compared the capacities of the MaSuRCA, SPAdes, and Unicycler assemblies in phylogenetic analyses of bacterial pathogens using both simulated and real reads. High consistency was observed between the reference genomes and hybrid assemblies for whole- (Figs. 1 and 2) and core-genome (Figs. 3 and 4) phylogeny of selected strains with both simulated and real reads. By estimating the phylogeny with both the reference genomes and hybrid assemblies, we observed that the MaSuRCA, SPAdes, and Unicycler assemblies produced a phylogenetic tree topology that was comparable with the reference genomes in all cases. The MaSuRCA, SPAdes, and Unicycler assemblies always clustered together with the reference genomes. The MaSuRCA, SPAdes, and Unicycler assemblies of *P. aeruginosa* PAO1 or *E. coli* O157:H7 Sakai with mediocre-quality long reads were on the same clade where those of low-quality long reads were located (Figs. 1 and 3). We thus demonstrate the potential of the hybrid assembly approaches of MaSuRCA, SPAdes, and Unicycler for accurate phylogenetic inference, as revealed by the congruent whole- and core-genome phylogenetic topology between the reference genomes and hybrid assemblies.

### Pan genomes

**Fig. 5:**
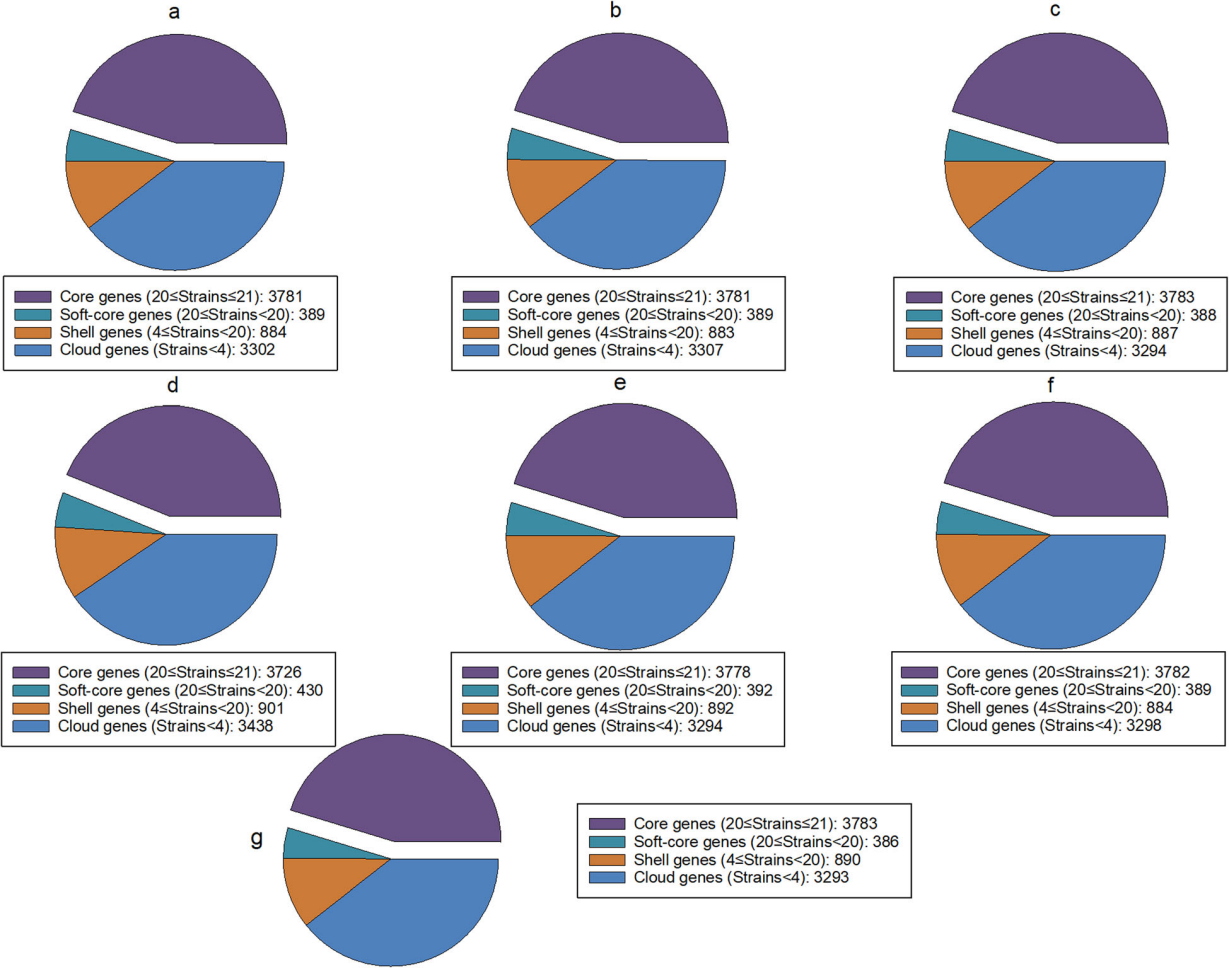
Pan genomes of the hybrid assemblies of *Salmonella* Typhimurium LT2 with simulated Illumina short reads and mediocre- or low-quality Oxford Nanopore long reads using MaSuRCA (mediocre-quality, **a** low-quality, **d**, SPAdes (mediocre-quality, **b** low-quality, **e**, and Unicycler (mediocre-quality, **c** low-quality, **f**) and 20 *S.* Typhimurium strains compared to the reference genome (**g**)

**Fig. 6:**
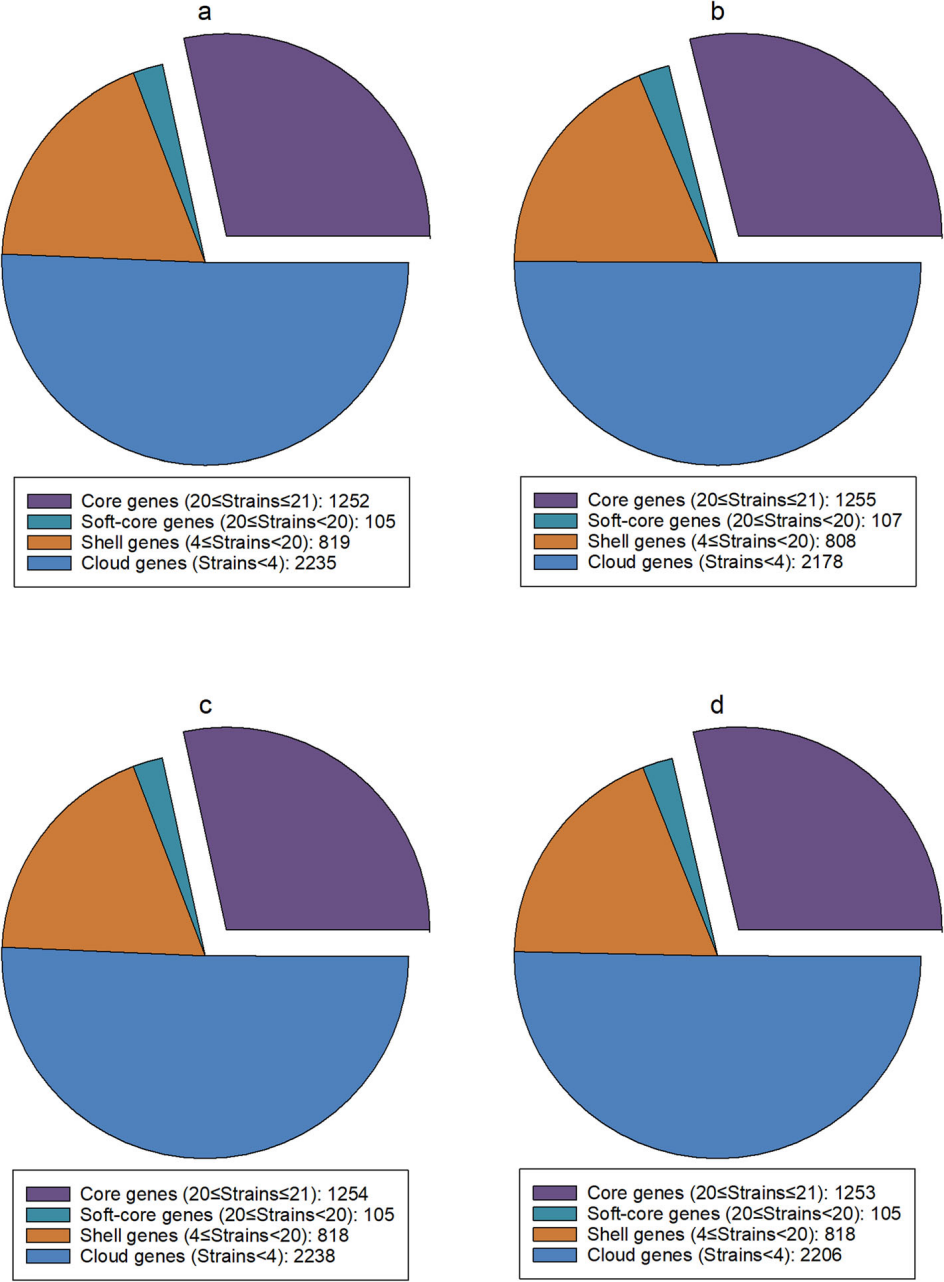
Pan genomes of the hybrid assemblies of *Campylobacter jejuni* CFSAN032806 with real Illumina short reads and Oxford Nanopore long reads using MaSuRCA (**a**), SPAdes (**b**), and Unicycler (**c**) and 20 *C. jejuni* strains compared to the reference genome (**d**)

The pan genomes of the MaSuRCA, SPAdes, and Unicycler assemblies of *S.* Typhimurium LT2 with mediocre-quality long reads were similar to that of the reference genome that had 8352 genes with 3783 core genes and 4569 accessory genes (Fig. 5). The hybrid assembly approaches of SPAdes and Unicycler tolerated a higher level of error in Oxford Nanopore long reads since the numbers of core and accessory genes of the pan genomes of the SPAdes and Unicycler assemblies of low-quality long reads were similar to those of the reference genome (Fig. 5). However, we observed a decrease in the number of core genes (3726) and an increase in the number of accessory genes (4769) in the pan genome of the MaSuRCA assembly of low-quality long reads compared to that of mediocre-quality long reads that had 3781 core genes and 4575 accessory genes. Our results thus demonstrate that MaSuRCA was less tolerant of a higher level of error rates in Oxford Nanopore long reads compared to SPAdes and Unicycler. The observed better performance of the SPAdes and Unicycler assemblies could be due to superior hybrid assembly processes where Illumina short reads can ameliorate the shortcomings of Oxford Nanopore long reads with errors that introduce truncated genes [6]. Our pan-genome analyses thus highlight the difficulty of MaSuRCA in using highly error-prone Oxford Nanopore long reads to produce accurate hybrid assemblies, which can lead to an imperfect representation of genome annotation. Noticeably, MaSuRCA, SPAdes, and Unicycler functioned well in the pan-genome analysis of *C. jejuni* CFSAN032806 with real reads, as the hybrid assemblies showed similar pan-genome compositions to the reference genome (Fig. 6). Goldstein et al. [18] also reported that the hybrid approaches of SPAdes and Unicycler dramatically improved the annotation of complex genomic features such as insertion sequences and secondary metabolite biosynthetic gene clusters of *Flavobacterium*, *Aeromonas*, and *Pseudonocardia* strains with real reads.

### Hybrid assembly approaches of MaSuRCA, SPAdes, and Unicycler

High-quality assemblies of bacterial pathogens are critical for all aspects of genomics, especially genome annotation and comparative genomics. Many bacterial genomic analyses greatly rely on finished genomes [31]. Although producing finished genomes remains prohibitive with the cost of finishing proportional to the number of gaps in the original assembly, it is clear that higher-quality assemblies, with long unbroken contigs, will have a positive impact on a wide range of genomic analyses of bacterial pathogens. We found that high-error Oxford Nanopore long reads can be efficiently assembled in combination with Illumina short reads to produce assemblies using the hybrid assembly pipeline of Unicycler, bringing us one step closer to the objective of “one chromosome, one contig” [19]. The result of the hybrid assembly approach of Unicycler is high-quality assemblies with fewer errors and gaps, which will enable more accurate downstream genomic analyses.

The hybrid assembly approach of MaSuRCA does not first produce an Illumina short-read graph [8]. Instead, it relies on a submodule of Flye for the final assembly of corrected mega-reads produced using both longer super-reads of Illumina short reads and Oxford Nanopore long reads [8, 11, 12]. As observed in our study, the one-step hybrid assembly algorithm of MaSuRCA resulted in more errors in the final assemblies. These biases became especially pronounced when low-quality long reads with a higher level of error were used. For the hybrid assembly approach of SPAdes, the set of Oxford Nanopore long reads are collected spanning the same pair of sink and source edges of the Illumina short-read assembly graph and close the coverage gap using the consensus sequence of all these reads. We found that although the SPAdes assemblies performed similarly to the Unicycler assemblies for genomic analyses, they were highly fragmented in all cases, which could be attributed to the fact that SPAdes does not assemble Oxford Nanopore long reads before gap closure. In contrast, after Unicycler produces the Illumina short-read graph, Oxford Nanopore long reads are then assembled with Miniasm, followed by multiple rounds of Racon polishing, for long-read bridging [9]. Senol Cali et al. [32] carried out a review to analyze state-of-the-art bioinformatic tools for Oxford Nanopore long reads in terms of accuracy, speed, memory efficiency, and scalability. After a comprehensive analysis, they recommended that Miniasm and Racon should be used for assembly and polishing, respectively. Moreover, Unicycler implements multiple rounds of Pilon polishing using Illumina short reads on the final assembly to further improve the sequence accuracy.

We can anticipate that with further development of Oxford Nanopore sequencing, long-read assembly quality will increase which could make hybrid assemblies produced by Unicycler more complete and accurate. Potential improvements to the hybrid assembly algorithm should include the assembly and polishing of Oxford Nanopore long reads, as well as the gap closure of the Illumina short-read assembly graph using Oxford Nanopore long reads. This is particularly important for repetitive and GC-rich regions that tend to be under-represented by Illumina sequencing.

## Conclusions

We benchmarked the hybrid assembly approaches of MaSuRCA, SPAdes, and Unicycler for genomic analyses of bacterial pathogens using Illumina and Oxford Nanopore sequencing. We used both simulated and real reads of bacterial strains spanning a wide range of genome sizes and GC contents. SPAdes and Unicycler produced more accurate hybrid assemblies of both simulated and real reads, and performed better in genomic analyses of AMR, virulence potential, and pan genome compared to MaSuRCA. However, despite the success of SPAdes, it has some obvious weaknesses that resulted in highly fragmented assemblies in all cases, whereas the MaSuRCA and Unicycler assemblies were more contiguous. The improvement of genomic analyses of bacterial pathogens was achieved by assembly algorithms that initiated the hybrid assembly with high-quality Illumina short reads and filled the gaps with Oxford Nanopore long reads. While improved contiguity was associated with the assembly of Oxford Nanopore long reads in advance of gap closure, Unicycler implemented both approaches and exhibited improved assemblies and genomic analyses, suggesting algorithmic approaches following that model may be most fruitful in the future.

Our research thus demonstrates that reference-grade hybrid assemblies of bacterial pathogens can be generated through the hybrid assembly pipeline of Unicycler using Illumina and Oxford Nanopore sequencing, with no manual intervention needed before and after assembly. However, we also observed that our Unicycler assemblies slightly diverged from the publicly available reference genomes (e.g. the small plasmid found in the Unicycler assembly of *S. aureus* CFSAN007894 that was missing in the PacBio-based reference genome), which could be due to either error in the original reference sequences or misassembly in the Unicycler assemblies. Hence, making comparisons for any given hybrid assembly approaches is, to some extent, demanding, even in the case where a reference genome is available. We chose to benchmark the hybrid assembly approaches of MaSuRCA, SPAdes, and Unicycler using their default parameters and recommended settings. Future optimization of these parameters and settings before implementation could further improve assembly algorithms. Meanwhile, we acknowledge that as sequencing technologies and assembly algorithms advance and mature, defining an optimal hybrid assembly approach for genomic analyses of bacterial pathogens is a continuous process. The extension of these algorithms to assemble larger genomes as well as assembly of metagenomes is an important area that requires dedicated studies to establish most appropriate algorithmic approaches for accurate results.

## Methods

### Simulated Illumina short reads and Oxford Nanopore long reads

Ten species of bacterial pathogens (Table 1) with ‘gold-standard’ reference genomes available from the National Center for Biotechnology Information (NCBI) Reference Sequence (RefSeq) Database (Additional file: Table S1) were selected, spanning a wide range of genome sizes and GC contents. We used ART 2.5.8 [33] to generate simulated Illumina paired-end short reads from each reference genome so as to mimic those from an Illumina MiSeq platform with a read length of 250 bp, mean fragment size of 400 bp, fragment size standard deviation of 60 bp, and coverage of 50 × .

To examine if hybrid assembly approaches could tolerate problems encountered in real error-prone Oxford Nanopore long reads, Badread 0.1.5 [15] was used based on the Nanopore error model to generate simulated Oxford Nanopore long reads of mediocre quality, defined as a read with a mean fragment size of 15,000 bp, fragment size standard deviation of 13,000 bp, mean identity of 85, max identity of 95, identity standard deviation of 5, and coverage of 50×. The chimera join rate, junk read rate, and random read rate of each simulated mediocre-quality dataset were set to 1%. Low-quality reads of each strain were also simulated by artificially introducing more chimeras, low-quality regions, and systematic basecalling errors. Simulation parameters of Badread were adjusted to mimic low-quality reads, with a glitch rate of 1000, glitch size of 100, glitch skip of 100, mean identity of 75, max identity of 90, identity standard deviation of 8, and coverage of 50×. The chimera join rate, junk read rate, and random read rate of each simulated low-quality dataset were adjusted to 10, 5, and 5%, respectively. Oxford Nanopore ligation adapters were added to the start and end of each read using Badread, with a start adapter rate of 90 and start adapter amount of 60, and an end adapter rate of 50 and end adapter amount of 20. Start and end adapter sequences were AATGTACTTCGTTCAGTTACGTATTGCT and GCAATACGTAACTGAACGAAGT, respectively.

### Real Illumina short reads and Oxford Nanopore long reads

Real Illumina short reads and Oxford Nanopore long reads of 12 strains of 11 species of bacterial pathogens (12 strains) (Table 2), together covering a wide range of genome sizes and GC contents, were obtained from the Sequence Read Archive (SRA) of the NCBI (Additional file: Table S2). PacBio assemblies serving as the reference genomes for strains with real reads were downloaded from the NCBI if they were publicly available. For strains with no available PacBio assemblies, PacBio long reads were assembled using the long-read assembly pipeline (normal mode) of Unicycler 0.4.8 [9], followed by three rounds of polishing with Illumina short reads using Pilon 1.23 [34].

### Hybrid assembly approaches of MaSuRCA, SPAdes, and Unicycler

Illumina short reads and Oxford Nanopore long reads of each strain were assembled using MaSuRCA 3.3.9, SPAdes 3.12.0, and Unicycler 0.4.8.

To perform the hybrid assembly using MaSuRCA, a configuration file was first created which contained assembly parameters. A shell script was then generated from the configuration file, which was executed to assemble the raw sequence data. The optimal *k*-mer size was automatically computed based on the data and GC content. Two passes of mega-reads were performed. The upper limit of jump coverage was set to down-sample the jumping library to 60× coverage. A recommended safe value of the jellyfish hash size was used, which was 20 times the estimated genome size.

For the hybrid assembly using SPAdes, paired-end libraries with forward-reverse (fr) orientation were provided, with *k*-mer sizes of 21, 33, and 55. Oxford Nanopore long reads were provided with the --nanopore option.

The normal mode was used for the hybrid assembly using Unicycler, which is intermediate regarding both contig size and misassembly rate. An Illumina short-read assembly graph was first produced using SPAdes, and then Miniasm and Racon were applied to build bridges with Oxford Nanopore long reads. Multiple rounds of short-read polishing were conducted using Pilon. Finally, circularized contigs were rotated to begin at a starting gene of *dnaA* or *repA* if one could be detected with BLAST+.

### Computational environments

The hybrid assembly using MaSuRCA was performed on the Linux operating system of Ubuntu 20.04 LTS on a computer with 12 threads of CPU and 16 GB of RAM. To avoid any performance variation caused by CPU overcommit, 10 threads of CPU were allocated in the option of the number of threads to use for MaSuRCA. SPAdes and Unicycler were available on the Amazon Web Services (AWS)-based GalaxyTrakr platform developed by the U.S. Food and Drug Administration (FDA) and intended for use by GenomeTrakr laboratories [35].

### Assessment of genome completeness and accuracy

Assembly quality was assessed using Quast 5.0.2 [36] by computing relavant metrics, including the number of contigs, total length (bp), and GC content. BUSCO 4.0.6 [37] was used to evaluate the genome completeness of each assembly based on the expected gene content of an assembly and length alignments to the BUSCO profiles, with 0.01 as the *E*-value cutoff for BLAST searches and three candidate regions to consider. The degree of genome completeness was expressed as complete, fragmented, and missing BUSCOs that represent the fractions of high-identity full-length genes, partially present genes, and absent genes, respectively. CSI Phylogeny 1.4 [38] was used to call SNPs of each hybrid assembly relative to the corresponding reference genome. Default settings were used, with 10× as the minimum depth at SNP positions, 10% as the minimum relative depth at SNP positions, 10 bp as the minimum distance between SNPs, 30 as the minimum SNP quality, 25 as the minimum read mapping quality, and 1.96 as the minimum Z-score. The number of SNPs was expressed as the number of SNPs per 1 million bp of the reference genome. To examine the similarity between each hybrid assembly and the corresponding reference genome, the orthologous average nucleotide identity (OrthoANI) value was determined by aligning the hybrid assembly to the reference genome using the ChunLab’s online Average Nucleotide Identity (ANI) calculator [39].

### Identifications of plasmids, ARGs, and virulence genes

Plasmids were identified using staramr 0.6.0 (https://github.com/phac-nml/staramr) against known plasmid sequences in the PlasmidFinder database [40], with 98% minimum identity and 60% minimum coverage. ARGs were detected using staramr 0.6.0 against known gene sequences in the ResFinder database [41], with 98% minimum identity and 60% minimum coverage. Virulence genes were identified using ABRicate 0.8.7 (https://github.com/tseemanN.A.bricate) incorporated with the Virulence Factors Database (VFDB) [42], with 80% minimum identity and 60% minimum coverage compared with known gene sequences.

### MLST

MLST was carried out using mlst 2.19.0 (https://github.com/tseemann/mlst) with integrated components of the PubMLST database [43] by scanning genomes against traditional PubMLST typing schemes based on seven housekeeping genes, with 95% minimum identity of full allelle to consider ‘similar’.

### Whole- and core-genome phylogenetic analyses

*Pseudomonas aeruginosa* PAO1 with simulated reads and *Listeria monocytogenes* CFSAN008100 with real reads were used for the whole-genome phylogenetic analyses. Thirty strains of *P. aeruginosa* (Additional file: Table S3) and *L. monocytogenes* (Additional file: Table S4) were included. CSI Phylogeny 1.4 [38] was utilized with the default settings, as previously described to call SNPs and infer phylogenetic relationship based on the concatenated alignment of the high-quality SNPs. *P. aeruginosa* DSM 50071 (RefSeq assembly accession: GCF_001045685.1) and *L. monocytogenes* EGD-e (RefSeq assembly accession: GCF_000196035.1) served as the reference genomes for *P. aeruginosa* PAO1 and *L. monocytogenes* CFSAN008100, respectively.

*Escherichia coli* O157:H7 Sakai with simulated reads and *Cronobacter sakazakii* CFSAN068773 with real reads were used for the core-genome phylogenetic analyses. Thirty strains of Shiga-toxin producing *E. coli* (STEC) (Additional file: Table S5) and *C. sakazakii* (Additional file: Table S6) were included. The core-genome SNP alignment was conducted using Parsnp 1.2 [44], allowing for automatic recruitment of the reference genome and requiring that all genomes be included in the final phylogeny.

The inferred whole- and core-genome phylogeny were visualized as a rectangular tree layout using Geneious Prime 2020.1.2. (Biomatters, Ltd., Auckland, New Zealand).

### Pan-genome analyses

*Salmonella* Typhimurium LT2 with simulated reads and *Campylobacter jejuni* CFSAN032806 with real reads were used for the pan-genome analyses. Twenty strains of *S.* Typhimurium (Additional file: Table S7) and *C. jejuni* (Additional file: Table S8) were included. To perform the pan-genome analyses, genome sequences were first annotated with Prokka 1.14.0 [45]. Pan genomes were then analyzed with Roary 3.12.0 [46] using the genome annotations as input to acquire the numbers of core, soft-core, shell, and cloud genomes.

### Statistical analyses

The Wilcoxon signed-rank test was performed using SigmaPlot 14.0 (Systat Software Inc., San Jose, CA) to assess whether there were significant differences (*P* < 0.05) among the reference genomes, MaSuRCA, SPAdes, and Unicycler assemblies in genome size, GC content, complete, fragmented, and missing BUSCOs, number of SNPs, OrthoANI values, and number of virulence genes.

## Supplementary information

**Additional file 1: Table S1.** Bacterial strains with simulated Illumina short reads and mediocre- or low-quality Oxford Nanopore long reads.

**Additional file 2: Table S2.** Bacterial strains with real Illumina short reads and Oxford Nanopore long reads.

**Additional file 3: Table S3.** Thirty strains of *Pseudomonas aeruginosa*.

**Additional file 4: Table S4.** Thirty strains of *Listeria monocytogenes*.

**Additional file 5: Table S5.** Thirty strains of Shiga-toxin producing *Escherichia coli*.

**Additional file 6: Table S6.** Thirty strains of *Cronobacter sakazakii*.

**Additional file 7: Table S7.** Twenty strains of *Salmonella* Typhimurium.

**Additional file 8: Table S8.** Twenty strains of *Campylobacter jejuni*.

**Additional file 9: Table S9.** Genome completeness of the hybrid assemblies of bacterial strains with simulated Illumina short reads and mediocre-quality Oxford Nanopore long reads using MaSuRCA, SPAdes, and Unicycler compared to their corresponding reference genomes.

**Additional file 10: Table S10.** Genome completeness of the hybrid assemblies of bacterial strains with simulated Illumina short reads and low-quality Oxford Nanopore long reads using MaSuRCA, SPAdes, and Unicycler compared to their corresponding reference genomes.

**Additional file 11: Table S11.** Genome completeness of the hybrid assemblies of bacterial strains with real Illumina short reads and Oxford Nanopore long reads using MaSuRCA, SPAdes, and Unicycler compared to their corresponding reference genomes.

**Additional file 12: Table S12.** Numbers of single nucleotide polymorphisms (SNPs) in the hybrid assemblies of bacterial strains with simulated Illumina short reads and mediocre-quality Oxford Nanopore long reads using MaSuRCA, SPAdes, and Unicycler, as determined by aligning to their corresponding reference genomes and expressed as SNPs per 1 million bp of the reference genome.

**Additional file 13: Table S13.** Numbers of single nucleotide polymorphisms (SNPs) in the hybrid assemblies of bacterial strains with simulated Illumina short reads and low-quality Oxford Nanopore long reads using MaSuRCA, SPAdes, and Unicycler, as determined by aligning to their corresponding reference genomes and expressed as SNPs per 1 million bp of the reference genome.

**Additional file 14: Table S14.** Numbers of single nucleotide polymorphisms (SNPs) in the hybrid assemblies of bacterial strains with real Illumina short reads and Oxford Nanopore long reads using MaSuRCA, SPAdes, and Unicycler, as determined by aligning to their corresponding reference genomes and expressed as SNPs per 1 million bp of the reference genome.

**Additional file 15: Table S15.** Average Nucleotide Identity (ANI) of the hybrid assemblies of bacterial strains with simulated Illumina short reads and mediocre-quality Oxford Nanopore long reads using MaSuRCA, SPAdes, and Unicycler, as determined by aligning to their corresponding reference genomes and expressed as OrthoANIu values (%).

**Additional file 16: Table S16.** Average Nucleotide Identity (ANI) of the hybrid assemblies of bacterial strains with simulated Illumina short reads and low-quality Oxford Nanopore long reads using MaSuRCA, SPAdes, and Unicycler, as determined by aligning to their corresponding reference genomes and expressed as OrthoANIu values (%).

**Additional file 17: Table S17.** Average Nucleotide Identity (ANI) of the hybrid assemblies of bacterial strains with real Illumina short reads and Oxford Nanopore long reads using MaSuRCA, SPAdes, and Unicycler, as determined by aligning to their corresponding reference genomes and expressed as OrthoANIu values (%).

**Additional file 18: Table S18.** Plasmids of bacterial strains with simulated Illumina short reads and mediocre-quality Oxford Nanopore long reads, as predicted based on their MaSuRCA, SPAdes, and Unicycler assemblies and compared to their corresponding reference genomes.

**Additional file 19: Table S19.** Plasmids of bacterial strains with simulated Illumina short reads and low-quality Oxford Nanopore long reads, as predicted based on their MaSuRCA, SPAdes, and Unicycler assemblies and compared to their corresponding reference genomes.

**Additional file 20: Table S20.** Plasmids of bacterial strains with real Illumina short reads and Oxford Nanopore long reads, as predicted based on their MaSuRCA, SPAdes, and Unicycler assemblies and compared to their corresponding reference genomes.

## Data Availability

All data generated or analyzed during this study are included in this published article and its supplementary information files.

## Abbreviations

AMR: Antimicrobial resistance
ANI: Average Nucleotide Identity
ARGs: Antimicrobial resistance genes
AWS: Amazon Web Services
BUSCOs: Benchmarking universal single-copy orthologs
ExPEC: Extraintestinal pathogenic *E. coli*
FDA: U.S. Food and Drug Administration
fr: Forward-reverse
IPEC: Intestinal pathogenic *E. coli*
NCBI: National Center for Biotechnology Information
NGS: Next-generation sequencing
OLC: Overlap-layout-consensus
OrthoANI: Orthologous average nucleotide identity
RefSeq: Reference Sequence
SNPs: Single nucleotide polymorphisms
SRA: Sequence Read Archive
STEC: Shiga-toxin producing *E. coli*
WGS: Whole-genome sequencing
VFDB: Virulence Factors Database
