# Supplementary material for: Benchmarking hybrid assembly approaches for genomic analyses of bacterial pathogens using Illumina and Oxford Nanopore sequencing
Source: BMC Genomics. 2020 Sep 14;21:631. doi: 10.1186/s12864-020-07041-8 (PMC7490894; doi:10.1186/s12864-020-07041-8)
Supplement: Supplementary file 1 — Additional file 1: Table S1. Bacterial strains with simulated Illumina short reads and mediocre- or low-quality Oxford Nanopore long reads. [file 12864_2020_7041_MOESM1_ESM.docx]

Table S1 Bacterial strains with simulated Illumina short reads and mediocre- or low-quality Oxford Nanopore long reads

| Strain | RefSeq assembly accession |
| --- | --- |
| *Pseudomonas aeruginosa* PAO1 | GCF_000006765.1 |
| *Escherichia coli* O157:H7 Sakai | GCF_000008865.2 |
| *Bacillus anthracis* Ames Ancestor | GCF_000008445.1 |
| *Klebsiella variicola* DSM 15968 | GCF_000828055.2 |
| *Salmonella* Typhimurium LT2 | GCF_000006945.2 |
| *Cronobacter sakazakii* ATCC 29544 | GCF_000982825.1 |
| *Clostridium botulinum* CDC_1632 | GCF_001889325.1 |
| *Listeria monocytogenes* EGD-e | GCF_000196035.1 |
| *Staphylococcus aureus* NCTC 8325 | GCF_000013425.1 |
| *Campylobacter jejuni* NCTC 11168 | GCF_000009085.1 |
