# Supplementary material for: Benchmarking hybrid assembly approaches for genomic analyses of bacterial pathogens using Illumina and Oxford Nanopore sequencing
Source: BMC Genomics. 2020 Sep 14;21:631. doi: 10.1186/s12864-020-07041-8 (PMC7490894; doi:10.1186/s12864-020-07041-8)
Supplement: Supplementary file 2 — Additional file 2: Table S2. Bacterial strains with real Illumina short reads and Oxford Nanopore long reads. [file 12864_2020_7041_MOESM2_ESM.docx]

Table S2 Bacterial strains with real Illumina short reads and Oxford Nanopore long reads

| Strain | Oxford Nanopore sequencing | | Illumina sequencing | | PacBio sequencing | |
| --- | --- | --- | --- | --- | --- | --- |
|  | Run accession | Platform | Run accession | Platform | Run accession | Platform |
| *Escherichia coli* O26:H11 CFSAN027350 | SRR8335315 | MinION | SRR8333590 | MiSeq | GCF_004358385.1 | RSII |
| *Escherichia coli* O26:H11 CFSAN027343 | SRR8335317 | MinION | SRR8333591 | MiSeq | GCF_004358405.1 | RSII |
| *Klebsiella variicola* CFSAN086180 | SRR10340802 | GridION | SRR9043694 | MiSeq | SRR8362625^a^ | RSII |
| *Klebsiella pneumonia* CFSAN086181 | SRR10340801 | GridION | SRR9042857 | MiSeq | SRR8362597 | RSII |
| *Salmonella* Bareilly CFSAN000189 | SRR10337242 | GridION | SRR9043663 | MiSeq | GCF_000439415.1 | RS |
| *Enterobacter cancerogenus* CFSAN086183 | SRR10340797 | GridION | SRR9043660 | MiSeq | SRR8362611 | RSII |
| *Citrobacter braakii* CFSAN086182 | SRR10340798 | GridION | SRR9043684 | MiSeq | SRR8362596 | RSII |
| *Cronobacter sakazakii* CFSAN068773 | SRR10340800 | GridION | SRR9043662 | MiSeq | SRR8362631 | RSII |
| *Listeria monocytogenes* CFSAN008100 | SRR10336618 | GridION | SRR9043686 | MiSeq | GCF_001005925.2 | RSII |
| *Staphylococcus aureus* CFSAN007894 | SRR10346110 | GridION | SRR9043687 | MiSeq | GCF_002633865.1 | RSII |
| *Campylobacter jejuni* CFSAN032806 | SRR10342325 | GridION | SRR9043691 | MiSeq | GCF_002407125.1 | RSII |
| *Campylobacter coli* CFSAN032805 | SRR10342326 | GridION | SRR9042864 | MiSeq | GCF_002407145.1 | RSII |

^a^If RefSeq assembly accession is not available from the National Center for Biotechnology Information (NCBI), PacBio run accession is provided.
