# Supplementary material for: Benchmarking hybrid assembly approaches for genomic analyses of bacterial pathogens using Illumina and Oxford Nanopore sequencing
Source: BMC Genomics. 2020 Sep 14;21:631. doi: 10.1186/s12864-020-07041-8 (PMC7490894; doi:10.1186/s12864-020-07041-8)
Supplement: Supplementary file 3 — Additional file 3: Table S3. Thirty strains of Pseudomonas aeruginosa. [file 12864_2020_7041_MOESM3_ESM.docx]

Table S3 Thirty strains of *Pseudomonas aeruginosa*

| Strain | RefSeq or GenBank assembly accession |
| --- | --- |
| 519119 | GCF_007559105.1 |
| AES1R | GCF_004355145.1 |
| AG1 | GCF_009662315.1 |
| AR_0110 | GCF_003204335.1 |
| AR_0230 | GCF_002968695.1 |
| AR439 | GCF_003073895.1 |
| C7-25 | GCF_902703215.1 |
| DK2 | GCF_000271365.1 |
| F30658 | GCF_001516265.1 |
| FDAARGOS_570 | GCF_003813025.1 |
| H27930 | GCF_001516325.2 |
| JB2 | GCF_003060845.1 |
| N15-01092 | GCF_003571505.1 |
| NCGM257 | GCF_001547955.1 |
| Ocean-1155 | GCF_002237405.1 |
| PA_D2 | GCF_001721765.1 |
| PA7 | GCF_000017205.1 |
| PA83 | GCF_002215345.1 |
| PA99 | GCA_009498355.1 |
| Pa127 | GCF_002205355.1 |
| PABL048 | GCF_003411785.2 |
| PB350 | GCF_002812905.2 |
| PB368 | GCF_002812845.1 |
| SCVJan | GCF_001900225.1 |
| T2436 | GCF_009720405.1 |
| T52373 | GCF_001516005.1 |
| UCBPP-PA14 | GCF_000014625.1 |
| VIT PC9 | GCA_010694505.1 |
| Y71 | GCF_003408495.1 |
| YL84 | GCF_000524595.1 |
