# Supplementary material for: Benchmarking hybrid assembly approaches for genomic analyses of bacterial pathogens using Illumina and Oxford Nanopore sequencing
Source: BMC Genomics. 2020 Sep 14;21:631. doi: 10.1186/s12864-020-07041-8 (PMC7490894; doi:10.1186/s12864-020-07041-8)
Supplement: Supplementary file 4 — Additional file 4: Table S4. Thirty strains of Listeria monocytogenes. [file 12864_2020_7041_MOESM4_ESM.docx]

Table S4 Thirty strains of *Listeria monocytogenes*

| Strain | RefSeq assembly accession |
| --- | --- |
| 4/52-1953 | GCF_010443035.1 |
| 08-6997 | GCF_000513635.1 |
| 10-092876-0145 LM9 | GCF_001999105.1 |
| 2018TE5305-1-4 | GCF_003144075.1 |
| 52859 | GCF_008807935.1 |
| AT3E | GCF_002557735.1 |
| AUSMDU00007774 | GCF_009664735.1 |
| CIIMS-NV-3 | GCF_003409075.1 |
| CIIMS-PH-1 | GCF_003433415.1 |
| Clip80459 | GCF_000026705.1 |
| FDA00006907 | GCF_002208215.1 |
| FSL R2-561 | GCF_000168575.2 |
| HCC23 | GCF_000021185.1 |
| L99 | GCF_000209755.1 |
| L1846 | GCF_001027085.1 |
| LMNC088 | GCF_900231475.1 |
| M13455 | GCF_003390475.1 |
| MF4562 | GCF_002848425.1 |
| MGYG-HGUT-02325 | GCF_902385845.1 |
| N1-011A | GCF_000438685.2 |
| N53-1 | GCF_000382945.1 |
| NCCP 14714 | GCF_004771175.1 |
| NCTC7973 | GCF_900637785.1 |
| NH1 | GCF_002969195.1 |
| PIR00546 | GCF_003031915.1 |
| PNUSAL000144 | GCF_002105675.1 |
| R479a | GCF_000613085.1 |
| ScottA | GCF_009866905.1 |
| SLCC2540 | GCF_000306905.1 |
| XYSN | GCF_002116675.1 |
