# Supplementary material for: Benchmarking hybrid assembly approaches for genomic analyses of bacterial pathogens using Illumina and Oxford Nanopore sequencing
Source: BMC Genomics. 2020 Sep 14;21:631. doi: 10.1186/s12864-020-07041-8 (PMC7490894; doi:10.1186/s12864-020-07041-8)
Supplement: Supplementary file 5 — Additional file 5: Table S5. Thirty strains of Shiga-toxin producing Escherichia coli. [file 12864_2020_7041_MOESM5_ESM.docx]

Table S5 Thirty strains of Shiga-toxin producing *Escherichia coli*

| Strain | RefSeq assembly accession |
| --- | --- |
| 143 | GCF_005221885.1 |
| 644-PT8 | GCF_001650295.1 |
| 2009EL-2071 | GCF_000299475.1 |
| 2011C-3274 | GCF_000703325.1 |
| 2159 | GCF_001753505.1 |
| 3384 | GCF_001753545.1 |
| 11128 | GCF_000010765.1 |
| 11368 | GCF_000091005.1 |
| 12009 | GCF_000010745.1 |
| AR-0428 | GCF_008727175.1 |
| EDL933 | GCF_000732965.1 |
| FDAARGOS_293 | GCF_002208865.2 |
| FRIK944 | GCF_001695515.1 |
| FWSEC0001 | GCF_005037725.1 |
| FWSEC0003 | GCF_005037845.1 |
| NIID070765 | GCF_003113515.1 |
| NIID080884 | GCF_003113735.1 |
| NIID112183 | GCF_003113835.1 |
| NIID121825 | GCF_003113555.1 |
| NIID132777 | GCF_003113195.1 |
| pv15-279 | GCF_003966795.1 |
| RM8385 | GCF_003112165.1 |
| RM8426 | GCF_003112185.1 |
| RM10386 | GCF_003112225.1 |
| RM12581 | GCF_000671295.1 |
| RM13514 | GCF_000520035.1 |
| Sakai substr. RIMD 0509952 | GCF_000008865.2 |
| SS17 | GCF_000730345.1 |
| TR01 | GCF_003722195.1 |
| TW14359 | GCF_000022225.1 |
