# Supplementary material for: Benchmarking hybrid assembly approaches for genomic analyses of bacterial pathogens using Illumina and Oxford Nanopore sequencing
Source: BMC Genomics. 2020 Sep 14;21:631. doi: 10.1186/s12864-020-07041-8 (PMC7490894; doi:10.1186/s12864-020-07041-8)
Supplement: Supplementary file 6 — Additional file 6: Table S6. Thirty strains of Cronobacter sakazakii. [file 12864_2020_7041_MOESM6_ESM.docx]

Table S6 Thirty strains of *Cronobacter sakazakii*

| Strain | RefSeq or GenBank assembly accession |
| --- | --- |
| 7G | GCF_002977495.1 |
| 468G | GCF_002977735.1 |
| 692 | GCF_001309295.1 |
| 2151 | GCF_000409265.1 |
| ATCC 29004 | GCF_003515895.1 |
| ATCC BAA-894 | GCF_000017665.1 |
| C7 | GCF_006861635.1 |
| cro780B3-2 | GCF_002977295.1 |
| cro911C2-2 | GCF_002094675.1 |
| CS-09 | GCA_003516105.2 |
| CS-58 | GCF_003207575.1 |
| E764 | GCF_000409245.1 |
| E772 | GCF_002199105.1 |
| ES15 | GCF_000263215.1 |
| ES35 | GCF_000409405.1 |
| ES713 | GCF_000409385.1 |
| GP1999 | GCF_002166885.1 |
| GZcsf-1 | GCF_003955925.1 |
| HA18070 | GCF_011605525.1 |
| HPB5174 | GCF_000698225.1 |
| M30 | GCF_001619915.1 |
| MGYG-HGUT-02460 | GCF_902387375.1 |
| MOD1_LR631 | GCF_002942225.1 |
| MOD1_LR707 | GCF_002942365.1 |
| MOD1_O26_4 | GCF_002974735.1 |
| MOD1-cr5 | GCF_002107915.1 |
| NBRC 102416 | GCF_000684935.1 |
| NCTC 8155 | GCF_001277275.1 |
| NM1240 | GCF_000974965.1 |
| S6 | GCF_003858315.1 |
