# Supplementary material for: Benchmarking hybrid assembly approaches for genomic analyses of bacterial pathogens using Illumina and Oxford Nanopore sequencing
Source: BMC Genomics. 2020 Sep 14;21:631. doi: 10.1186/s12864-020-07041-8 (PMC7490894; doi:10.1186/s12864-020-07041-8)
Supplement: Supplementary file 7 — Additional file 7: Table S7. Twenty strains of Salmonella Typhimurium. [file 12864_2020_7041_MOESM7_ESM.docx]

Table S7 Twenty strains of *Salmonella* Typhimurium

| Strain | RefSeq assembly accession |
| --- | --- |
| 01ST04081 | GCF_006384995.1 |
| 798 | GCF_000252875.1 |
| 14028S | GCF_000022165.1 |
| 138736 | GCF_000636135.1 |
| AUSMDU00008979 | GCF_009664635.1 |
| CFSAN001921 | GCF_000430145.2 |
| D23580 | GCF_000027025.1 |
| FDAARGOS_317 | GCF_002984285.1 |
| FORC098 | GCF_007431545.1 |
| L-3553 | GCF_000828595.1 |
| NCCP 16207 | GCF_009884375.1 |
| PNCS014862 | GCF_004919365.1 |
| RM10961 | GCF_001922185.1 |
| SAP17-7399 | GCF_005885855.1 |
| sg_wt7 | GCF_004194625.2 |
| ST90 | GCF_012051625.1 |
| U288 | GCF_000380325.1 |
| USDA-ARS-USMARC-1808 | GCF_001623725.1 |
| WW012 | GCF_002313125.1 |
| YU39 | GCF_001006525.1 |
