# Supplementary material for: Benchmarking hybrid assembly approaches for genomic analyses of bacterial pathogens using Illumina and Oxford Nanopore sequencing
Source: BMC Genomics. 2020 Sep 14;21:631. doi: 10.1186/s12864-020-07041-8 (PMC7490894; doi:10.1186/s12864-020-07041-8)
Supplement: Supplementary file 8 — Additional file 8: Table S8. Twenty strains of Campylobacter jejuni. [file 12864_2020_7041_MOESM8_ESM.docx]

Table S8 Twenty strains of *Campylobacter jejuni*

| Strain | RefSeq or GenBank assembly accession |
| --- | --- |
| 00-0949 | GCF_000835365.1 |
| 11168H/araE | GCF_002238375.1 |
| 12567 | GCF_003060725.1 |
| 32488 | GCF_000430385.1 |
| AR-0414 | GCF_008727435.1 |
| CFSAN096345 | GCF_003956045.1 |
| CJ018CCUA | GCF_003368225.1 |
| F38011 | GCF_000772225.1 |
| HF5-4A-4 | GCF_001951235.1 |
| HPC5 | GCF_003574945.1 |
| I2019004398 | GCA_005211465.1 |
| I2019005837 | GCA_007740315.1 |
| MTVDSCj16 | GCF_001721985.1 |
| NADC 20827 | GCF_009498375.1 |
| NS4-5-1 | GCF_001951315.1 |
| PNUSAC014798 | GCA_010360725.1 |
| SCJK2 | GCF_007431745.1 |
| YH002 | GCF_002101355.1 |
| ZP3204 | GCF_001870105.1 |
| ZS005 | GCF_012224585.1 |
