# Supplementary material for: Benchmarking hybrid assembly approaches for genomic analyses of bacterial pathogens using Illumina and Oxford Nanopore sequencing
Source: BMC Genomics. 2020 Sep 14;21:631. doi: 10.1186/s12864-020-07041-8 (PMC7490894; doi:10.1186/s12864-020-07041-8)
Supplement: Supplementary file 9 — Additional file 9: Table S9. Genome completeness of the hybrid assemblies of bacterial strains with simulated Illumina short reads and mediocre-quality Oxford Nanopore long reads using MaSuRCA, SPAdes, and Unicycler compared to their corresponding reference genomes. [file 12864_2020_7041_MOESM9_ESM.docx]

Table S9 Genome completeness of the hybrid assemblies of bacterial strains with simulated Illumina short reads and mediocre-quality Oxford Nanopore long reads using MaSuRCA, SPAdes, and Unicycler compared to their corresponding reference genomes

| Strain | Complete BUSCOs (%) | | | | Fragmented BUSCOs (%) | | | | Missing BUSCOs (%) | | | |
| --- | --- | --- | --- | --- | --- | --- | --- | --- | --- | --- | --- | --- |
|  | MaSuRCA | SPAdes | Unicycler | Reference | MaSuRCA | SPAdes | Unicycler | Reference | MaSuRCA | SPAdes | Unicycler | Reference |
| *Pseudomonas aeruginosa* PAO1 | 100.0 | 100.0 | 100.0 | 100.0 | 0.0 | 0.0 | 0.0 | 0.0 | 0.0 | 0.0 | 0.0 | 0.0 |
| *Escherichia coli* O157:H7 Sakai | 100.0 | 100.0 | 100.0 | 100.0 | 0.0 | 0.0 | 0.0 | 0.0 | 0.0 | 0.0 | 0.0 | 0.0 |
| *Bacillus anthracis* Ames Ancestor | 99.3 | 99.3 | 98.6 | 99.3 | 0.0 | 0.0 | 0.0 | 0.0 | 0.7 | 0.7 | 1.4 | 0.7 |
| *Klebsiella variicola* DSM 15968 | 100.0 | 100.0 | 100.0 | 100.0 | 0.0 | 0.0 | 0.0 | 0.0 | 0.0 | 0.0 | 0.0 | 0.0 |
| *Salmonella* Typhimurium LT2 | 99.3 | 99.3 | 99.3 | 99.3 | 0.0 | 0.0 | 0.0 | 0.0 | 0.7 | 0.7 | 0.7 | 0.7 |
| *Cronobacter sakazakii* ATCC 29544 | 100.0 | 100.0 | 100.0 | 100.0 | 0.0 | 0.0 | 0.0 | 0.0 | 0.0 | 0.0 | 0.0 | 0.0 |
| *Clostridium botulinum* CDC_1632 | 99.3 | 99.3 | 99.3 | 99.3 | 0.0 | 0.0 | 0.0 | 0.0 | 0.7 | 0.7 | 0.7 | 0.7 |
| *Listeria monocytogenes* EGD-e | 100.0 | 100.0 | 100.0 | 100.0 | 0.0 | 0.0 | 0.0 | 0.0 | 0.0 | 0.0 | 0.0 | 0.0 |
| *Staphylococcus aureus* NCTC 8325 | 98.0 | 98.0 | 98.0 | 98.0 | 1.4 | 1.4 | 1.4 | 1.4 | 0.6 | 0.6 | 0.6 | 0.6 |
| *Campylobacter jejuni* NCTC 11168 | 88.5 | 88.5 | 88.5 | 88.5 | 0.7 | 0.7 | 0.7 | 0.7 | 10.8 | 10.8 | 10.8 | 10.8 |
| Average | 98.4 | 98.4 | 98.4 | 98.4 | 0.2 | 0.2 | 0.2 | 0.2 | 1.4 | 1.4 | 1.4 | 1.4 |
