# Supplementary material for: Benchmarking hybrid assembly approaches for genomic analyses of bacterial pathogens using Illumina and Oxford Nanopore sequencing
Source: BMC Genomics. 2020 Sep 14;21:631. doi: 10.1186/s12864-020-07041-8 (PMC7490894; doi:10.1186/s12864-020-07041-8)
Supplement: Supplementary file 11 — Additional file 11: Table S11. Genome completeness of the hybrid assemblies of bacterial strains with real Illumina short reads and Oxford Nanopore long reads using MaSuRCA, SPAdes, and Unicycler compared to their corresponding reference genomes. [file 12864_2020_7041_MOESM11_ESM.docx]

Table S11 Genome completeness of the hybrid assemblies of bacterial strains with real Illumina short reads and Oxford Nanopore long reads using MaSuRCA, SPAdes, and Unicycler compared to their corresponding reference genomes

| Strain | Complete BUSCOs (%) | | | | Fragmented BUSCOs (%) | | | | Missing BUSCOs (%) | | | |
| --- | --- | --- | --- | --- | --- | --- | --- | --- | --- | --- | --- | --- |
|  | MaSuRCA | SPAdes | Unicycler | Reference | MaSuRCA | SPAdes | Unicycler | Reference | MaSuRCA | SPAdes | Unicycler | Reference |
| *Escherichia coli* O26:H11 CFSAN027343 | 100.0 | 100.0 | 100.0 | 100.0 | 0.0 | 0.0 | 0.0 | 0.0 | 0.0 | 0.0 | 0.0 | 0.0 |
| *Escherichia coli* O26:H11 CFSAN027350 | 100.0 | 100.0 | 100.0 | 100.0 | 0.0 | 0.0 | 0.0 | 0.0 | 0.0 | 0.0 | 0.0 | 0.0 |
| *Klebsiella variicola* CFSAN086180 | 100.0 | 100.0 | 100.0 | 100.0 | 0.0 | 0.0 | 0.0 | 0.0 | 0.0 | 0.0 | 0.0 | 0.0 |
| *Klebsiella pneumoniae* CFSAN086181 | 100.0 | 100.0 | 100.0 | 100.0 | 0.0 | 0.0 | 0.0 | 0.0 | 0.0 | 0.0 | 0.0 | 0.0 |
| *Enterobacter cancerogenus* CFSAN086183 | 99.3 | 99.3 | 99.3 | 99.3 | 0.0 | 0.0 | 0.0 | 0.0 | 0.7 | 0.7 | 0.7 | 0.7 |
| *Salmonella* Bareilly CFSAN000189 | 99.3 | 99.3 | 99.3 | 99.3 | 0.0 | 0.0 | 0.0 | 0.0 | 0.7 | 0.7 | 0.7 | 0.7 |
| *Citrobacter braakii* CFSAN086182 | 98.6 | 98.6 | 98.6 | 98.6 | 0.7 | 0.7 | 0.7 | 0.7 | 0.7 | 0.7 | 0.7 | 0.7 |
| *Cronobacter sakazakii* CFSAN068773 | 100.0 | 100.0 | 100.0 | 100.0 | 0.0 | 0.0 | 0.0 | 0.0 | 0.0 | 0.0 | 0.0 | 0.0 |
| *Listeria monocytogenes* CFSAN008100 | 100.0 | 100.0 | 100.0 | 100.0 | 0.0 | 0.0 | 0.0 | 0.0 | 0.0 | 0.0 | 0.0 | 0.0 |
| *Staphylococcus aureus* CFSAN007894 | 100.0 | 100.0 | 100.0 | 100.0 | 0.0 | 0.0 | 0.0 | 0.0 | 0.0 | 0.0 | 0.0 | 0.0 |
| *Campylobacter jejuni* CFSAN032806 | 84.5 | 85.8 | 85.8 | 85.8 | 1.4 | 1.4 | 1.4 | 1.4 | 14.1 | 12.8 | 12.8 | 12.8 |
| *Campylobacter coli* CFSAN032805 | 83.1 | 84.5 | 84.5 | 84.5 | 4.1 | 3.4 | 3.4 | 3.4 | 12.8 | 12.1 | 12.1 | 12.1 |
| Average | 97.1 | 97.3 | 97.3 | 97.3 | 0.5 | 0.5 | 0.5 | 0.5 | 2.4 | 2.3 | 2.3 | 2.3 |
