# Supplementary material for: Benchmarking hybrid assembly approaches for genomic analyses of bacterial pathogens using Illumina and Oxford Nanopore sequencing
Source: BMC Genomics. 2020 Sep 14;21:631. doi: 10.1186/s12864-020-07041-8 (PMC7490894; doi:10.1186/s12864-020-07041-8)
Supplement: Supplementary file 13 — Additional file 13: Table S13. Numbers of single nucleotide polymorphisms (SNPs) in the hybrid assemblies of bacterial strains with simulated Illumina short reads and low-quality Oxford Nanopore long reads using MaSuRCA, SPAdes, and Unicycler, as determined by aligning to their corresponding reference genomes and expressed as SNPs per 1 million bp of the reference genome. [file 12864_2020_7041_MOESM13_ESM.docx]

Table S13 Numbers of single nucleotide polymorphisms (SNPs) in the hybrid assemblies of bacterial strains with simulated Illumina short reads and low-quality Oxford Nanopore long reads using MaSuRCA, SPAdes, and Unicycler, as determined by aligning to their corresponding reference genomes and expressed as SNPs per one million bp of the reference genome

| Strain | Number of SNPs | | |
| --- | --- | --- | --- |
|  | MaSuRCA | SPAdes | Unicycler |
| *Pseudomonas aeruginosa* PAO1 | 3.67 | 0.16 | 1.12 |
| *Escherichia coli* O157:H7 Sakai | 1.97 | 2.32 | 0.00 |
| *Bacillus anthracis* Ames Ancestor | 2.18 | 0.00 | 0.00 |
| *Klebsiella variicola* DSM 15968 | 2.17 | 0.18 | 0.91 |
| *Salmonella* Typhimurium LT2 | 2.42 | 0.20 | 0.00 |
| *Cronobacter sakazakii* ATCC 29544 | 3.65 | 1.07 | 0.00 |
| *Clostridium botulinum* CDC_1632 | 2.96 | 4.55 | 0.46 |
| *Listeria monocytogenes* EGD-e | 1.36 | 0.00 | 0.00 |
| *Staphylococcus aureus* NCTC 8325 | 0.71 | 3.54 | 0.71 |
| *Campylobacter jejuni* NCTC 11168 | 4.26 | 2.44 | 0.00 |
| Average | 2.54 | 1.45 | 0.32 |
