# Supplementary material for: Benchmarking hybrid assembly approaches for genomic analyses of bacterial pathogens using Illumina and Oxford Nanopore sequencing
Source: BMC Genomics. 2020 Sep 14;21:631. doi: 10.1186/s12864-020-07041-8 (PMC7490894; doi:10.1186/s12864-020-07041-8)
Supplement: Supplementary file 14 — Additional file 14: Table S14. Numbers of single nucleotide polymorphisms (SNPs) in the hybrid assemblies of bacterial strains with real Illumina short reads and Oxford Nanopore long reads using MaSuRCA, SPAdes, and Unicycler, as determined by aligning to their corresponding reference genomes and expressed as SNPs per 1 million bp of the reference genome. [file 12864_2020_7041_MOESM14_ESM.docx]

Table S14 Numbers of single nucleotide polymorphisms (SNPs) in the hybrid assemblies of bacterial strains with real Illumina short reads and Oxford Nanopore long reads using MaSuRCA, SPAdes, and Unicycler, as determined by aligning to their corresponding reference genomes and expressed as SNPs per one million bp of the reference genome

| Strain | Number of SNPs | | |
| --- | --- | --- | --- |
|  | MaSuRCA | SPAdes | Unicycler |
| *Escherichia coli* O26:H11 CFSAN027343 | 4.85 | 0.69 | 1.90 |
| *Escherichia coli* O26:H11 CFSAN027350 | 6.79 | 1.25 | 0.72 |
| *Klebsiella variicola* CFSAN086180 | 0.18 | 0.18 | 0.00 |
| *Klebsiella pneumoniae* CFSAN086181 | 0.38 | 0.96 | 0.00 |
| *Enterobacter cancerogenus* CFSAN086183 | 2.20 | 1.40 | 0.00 |
| *Salmonella* Bareilly CFSAN000189 | 0.00 | 0.42 | 0.00 |
| *Citrobacter braakii* CFSAN086182 | 0.20 | 0.00 | 0.00 |
| *Cronobacter sakazakii* CFSAN068773 | 0.87 | 0.00 | 0.00 |
| *Listeria monocytogenes* CFSAN008100 | 21.56 | 0.00 | 0.97 |
| *Staphylococcus aureus* CFSAN007894 | 1.81 | 3.26 | 0.36 |
| *Campylobacter jejuni* CFSAN032806 | 5.05 | 1.12 | 0.56 |
| *Campylobacter coli* CFSAN032805 | 4.00 | 0.57 | 2.29 |
| Average | 3.99 | 0.82 | 0.57 |
