# Supplementary material for: Benchmarking hybrid assembly approaches for genomic analyses of bacterial pathogens using Illumina and Oxford Nanopore sequencing
Source: BMC Genomics. 2020 Sep 14;21:631. doi: 10.1186/s12864-020-07041-8 (PMC7490894; doi:10.1186/s12864-020-07041-8)
Supplement: Supplementary file 15 — Additional file 15: Table S15. Average Nucleotide Identity (ANI) of the hybrid assemblies of bacterial strains with simulated Illumina short reads and mediocre-quality Oxford Nanopore long reads using MaSuRCA, SPAdes, and Unicycler, as determined by aligning to their corresponding reference genomes and expressed as OrthoANIu values (%). [file 12864_2020_7041_MOESM15_ESM.docx]

Table S15 Average Nucleotide Identity (ANI) of the hybrid assemblies of bacterial strains with simulated Illumina short reads and mediocre-quality Oxford Nanopore long reads using MaSuRCA, SPAdes, and Unicycler, as determined by aligning to their corresponding reference genomes and expressed as OrthoANIu values (%)

| Strain | OrthoANIu value (%) | | |
| --- | --- | --- | --- |
|  | MaSuRCA | SPAdes | Unicycler |
| *Pseudomonas aeruginosa* PAO1 | 99.99 | 99.97 | 99.98 |
| *Escherichia coli* O157:H7 Sakai | 100.00 | 99.97 | 99.99 |
| *Bacillus anthracis* Ames Ancestor | 99.99 | 99.97 | 99.98 |
| *Klebsiella variicola* DSM 15968 | 99.99 | 99.98 | 99.99 |
| *Salmonella* Typhimurium LT2 | 99.99 | 99.96 | 99.99 |
| *Cronobacter sakazakii* ATCC 29544 | 99.96 | 99.95 | 99.98 |
| *Clostridium botulinum* CDC_1632 | 99.96 | 99.96 | 99.96 |
| *Listeria monocytogenes* EGD-e | 99.99 | 99.99 | 99.99 |
| *Staphylococcus aureus* NCTC 8325 | 99.97 | 99.95 | 99.97 |
| *Campylobacter jejuni* NCTC 11168 | 99.99 | 100.00 | 99.98 |
| Average | 99.98 | 99.97 | 99.98 |
