# Supplementary material for: Benchmarking hybrid assembly approaches for genomic analyses of bacterial pathogens using Illumina and Oxford Nanopore sequencing
Source: BMC Genomics. 2020 Sep 14;21:631. doi: 10.1186/s12864-020-07041-8 (PMC7490894; doi:10.1186/s12864-020-07041-8)
Supplement: Supplementary file 17 — Additional file 17: Table S17. Average Nucleotide Identity (ANI) of the hybrid assemblies of bacterial strains with real Illumina short reads and Oxford Nanopore long reads using MaSuRCA, SPAdes, and Unicycler, as determined by aligning to their corresponding reference genomes and expressed as OrthoANIu values (%). [file 12864_2020_7041_MOESM17_ESM.docx]

Table S17 Average Nucleotide Identity (ANI) of the hybrid assemblies of bacterial strains with real Illumina short reads and Oxford Nanopore long reads using MaSuRCA, SPAdes, and Unicycler, as determined by aligning to their corresponding reference genomes and expressed as OrthoANIu values (%)

| Strain | OrthoANIu value (%) | | |
| --- | --- | --- | --- |
|  | MaSuRCA | SPAdes | Unicycler |
| *Escherichia coli* O26:H11 CFSAN027343 | 99.91 | 99.95 | 99.91 |
| *Escherichia coli* O26:H11 CFSAN027350 | 99.95 | 99.96 | 99.95 |
| *Klebsiella variicola* CFSAN086180 | 99.99 | 99.99 | 99.98 |
| *Klebsiella pneumoniae* CFSAN086181 | 99.97 | 99.98 | 100.00 |
| *Enterobacter cancerogenus* CFSAN086183 | 99.97 | 99.99 | 100.00 |
| *Salmonella* Bareilly CFSAN000189 | 99.97 | 99.99 | 99.99 |
| *Citrobacter braakii* CFSAN086182 | 99.99 | 100.00 | 100.00 |
| *Cronobacter sakazakii* CFSAN068773 | 99.97 | 99.98 | 100.00 |
| *Listeria monocytogenes* CFSAN008100 | 99.98 | 99.99 | 99.98 |
| *Staphylococcus aureus* CFSAN007894 | 99.95 | 99.95 | 99.87 |
| *Campylobacter jejuni* CFSAN032806 | 99.99 | 99.98 | 99.98 |
| *Campylobacter coli* CFSAN032805 | 99.97 | 99.99 | 99.99 |
| Average | 99.97 | 99.98 | 99.97 |
