# Supplementary material for: Benchmarking hybrid assembly approaches for genomic analyses of bacterial pathogens using Illumina and Oxford Nanopore sequencing
Source: BMC Genomics. 2020 Sep 14;21:631. doi: 10.1186/s12864-020-07041-8 (PMC7490894; doi:10.1186/s12864-020-07041-8)
Supplement: Supplementary file 18 — Additional file 18: Table S18. Plasmids of bacterial strains with simulated Illumina short reads and mediocre-quality Oxford Nanopore long reads, as predicted based on their MaSuRCA, SPAdes, and Unicycler assemblies and compared to their corresponding reference genomes. [file 12864_2020_7041_MOESM18_ESM.docx]

Table S18 Plasmids of bacterial strains with simulated Illumina short reads and mediocre-quality Oxford Nanopore long reads, as predicted based on their MaSuRCA, SPAdes, and Unicycler assemblies and compared to their corresponding reference genomes^a^

| Strain | Plasmid | | | |
| --- | --- | --- | --- | --- |
|  | MaSuRCA | SPAdes | Unicycler | Reference |
| *Escherichia coli* O157:H7 Sakai | IncFIB (AP001918) | IncFIB (AP001918) | IncFIB (AP001918) | IncFIB (AP001918) |
| *Bacillus anthracis* Ames Ancestor | rep3 | rep3 | rep3 | rep3 |
| *Salmonella* Typhimurium LT2 | IncFIB (S)  IncFII (S) | IncFIB (S)  IncFII (S) | IncFIB (S) | IncFIB (S)  IncFII (S) |
| *Cronobacter sakazakii* ATCC 29544 | IncFIB (pCTU3) | IncFIB (pCTU3) | IncFIB (pCTU3) | IncFIB (pCTU3) |
| *Staphylococcus aureus* NCTC 8325 | rep7 | rep7 | rep7 | rep7 |

^a^No plasmids were detected in *P. aeruginosa* PAO1, *K. variicola* DSM 15968, *L. monocytogenes* EGD-e, or *C. jejuni* NCTC 11168, as predicted based on the reference genomes and hybrid assemblies.
