# Supplementary material for: Benchmarking hybrid assembly approaches for genomic analyses of bacterial pathogens using Illumina and Oxford Nanopore sequencing
Source: BMC Genomics. 2020 Sep 14;21:631. doi: 10.1186/s12864-020-07041-8 (PMC7490894; doi:10.1186/s12864-020-07041-8)
Supplement: Supplementary file 20 — Additional file 20: Table S20. Plasmids of bacterial strains with real Illumina short reads and Oxford Nanopore long reads, as predicted based on their MaSuRCA, SPAdes, and Unicycler assemblies and compared to their corresponding reference genomes. [file 12864_2020_7041_MOESM20_ESM.docx]

Table S20 Plasmids of bacterial strains with real Illumina short reads and Oxford Nanopore long reads, as predicted based on their MaSuRCA, SPAdes, and Unicycler assemblies and compared to their corresponding reference genomes^a^

| Strain | Plasmid | | | |
| --- | --- | --- | --- | --- |
|  | MaSuRCA | SPAdes | Unicycler | Reference |
| *Escherichia coli* O26:H11 CFSAN027343 | IncB/O/K/Z | IncB/O/K/Z | N.D.^b^ | IncB/O/K/Z |
| *Escherichia coli* O26:H11 CFSAN027350 | IncFIB (AP001918) | IncFIB (AP001918) | IncFIB (AP001918) | IncFIB (AP001918) |
| *Salmonella* Bareilly CFSAN000189 | IncFII(S) | IncFII(S) | IncFII(S) | IncFII(S) |
| *Staphylococcus aureus* CFSAN007894 | rep20 | rep20 | rep20 | rep20 |

^a^No plasmids were detected in *K. variicola* CFSAN086180, *K. pneumoniae* CFSAN086181, *E. cancerogenus* CFSAN086183, *C. braakii* CFSAN086182, *C. sakazakii* CFSAN068773, *L. monocytogenes* CFSAN008100, *C. jejuni* CFSAN032806, or *C. coli* CFSAN032805, as predicted based on the reference genomes and hybrid assemblies.

^b^N.D., not detected.
